# Temporary adhesion of the proseriate flatworm *Minona ileanae*

**DOI:** 10.1098/rstb.2019.0194

**Published:** 2019-09-09

**Authors:** Robert Pjeta, Julia Wunderer, Philip Bertemes, Teresa Hofer, Willi Salvenmoser, Birgit Lengerer, Stefan Coassin, Gertraud Erhart, Christian Beisel, Daniel Sobral, Leopold Kremser, Herbert Lindner, Marco Curini-Galletti, Claus-Peter Stelzer, Michael W. Hess, Peter Ladurner

**Affiliations:** 1 Institute of Zoology and Center of Molecular Biosciences Innsbruck, University of Innsbruck, 6020 Innsbruck, Austria; 2 Biology of Marine Organisms and Biomimetics, Research Institute for Biosciences, University of Mons, 7000 Mons, Belgium; 3 Division of Genetic Epidemiology, Department of Medical Genetics, Molecular and Clinical Pharmacology, Medical University of Innsbruck, 6020 Innsbruck, Austria; 4 Division of Clinical Biochemistry, Biocenter, Medical University of Innsbruck, 6020 Innsbruck, Austria; 5 Division of Histology and Embryology, Medical University of Innsbruck, 6020 Innsbruck, Austria; 6 Department of Biosystems Science and Engineering, ETH Zürich, Basel, Switzerland; 7 Instituto Gulbenkian de Ciência, Oeiras, Portugal; 8 Dipartimento di Medicina Veterinaria, Università di Sassari, 07100 Sassari, Italy; 9 Research Institute for Limnology, University of Innsbruck, 5310 Mondsee, Austria

**Keywords:** bioadhesion, Platyhelminthes, flatworms, *Planaria*

## Abstract

Flatworms can very rapidly attach to and detach from many substrates. In the presented work, we analysed the adhesive system of the marine proseriate flatworm *Minona ileanae*. We used light-, scanning- and transmission electron microscopy to analyse the morphology of the adhesive organs, which are located at the ventral side of the tail-plate. We performed transcriptome sequencing and differential RNA-seq for the identification of tail-specific transcripts. Using *in situ* hybridization expression screening, we identified nine transcripts that were expressed in the cells of the adhesive organs. Knock-down of five of these transcripts by RNA interference led to a reduction of the animal's attachment capacity. Adhesive proteins in footprints were confirmed using mass spectrometry and antibody staining. Additionally, lectin labelling of footprints revealed the presence of several sugar moieties. Furthermore, we determined a genome size of about 560 Mb for *M. ileanae*. We demonstrated the potential of Oxford Nanopore sequencing of genomic DNA as a cost-effective tool for identifying the number of repeats within an adhesive protein and for combining transcripts that were fragments of larger genes. A better understanding of the molecules involved in flatworm bioadhesion can pave the way towards developing innovative glues with reversible adhesive properties.

This article is part of the theme issue ‘Transdisciplinary approaches to the study of adhesion and adhesives in biological systems’.

## 1. Introduction

The adhesion of animals to a surface is a common phenomenon in nature [1,2]. Mussels, barnacles and ascidians attach to the substrate as larvae by secreting adhesive substances and remain permanently attached throughout their lifetime. In recent years significant progress has been made in understanding permanent adhesion, mostly by studies on mussels, tubeworms and barnacles [3–5]. The molecules involved in permanent adhesion have been elucidated, and the characterization of the adhesives has led to the generation of biomimetic glues [6,7]. In contrast to permanent adhesion, echinoderms, *Hydra* and flatworms use a temporary adhesive system for functions such as attachment, locomotion, feeding and defence. Their temporary adhesion relies on the secretion of an adhesive material that, upon detachment of the animal, stays behind permanently on the substrate as a so-called footprint [8–12]. For *Hydra* and the sea star *Asterias rubens*, a collection of foot-specific adhesion candidate proteins have been identified, but the true glue proteins remain to be elucidated [8,11]. In *A. rubens*, one footprint-specific cohesive protein has been identified and further characterized [9]. Recent progress has been made in understanding attachment and release in the marine flatworm *Macrostomum lignano* [12]. The adhesive mainly consists of two large proteins, namely Mlig-ap1 and Mlig-ap2. Interestingly, the proposed cohesion protein of *M. lignano*, Mlig-ap1, shares similar protein domains with the sea star cohesion protein [12]. However, details on the protein domains responsible for attachment, the mechanism of Mlig-ap1 and Mlig-ap2 interaction, the role of carbohydrates, and the nature of the release molecule remain to be examined. In addition, flatworm adhesive organs display great diversity with respect to their morphology [13]. Notably, no homologue of Mlig-ap2 can be found in the National Center for Biotechnology Information (NCBI) database, which comprises 10.921 Taxonomy ID entries for Platyhelminthes (Taxonomy ID 6157). This lack of homologues raises questions about adhesive molecules of other flatworm species. Thus far, even less is known regarding the detachment mechanisms used for temporary adhesion [13–15].

In this study, we analysed the adhesive system of the proseriate flatworm *Minona ileanae*. We examined the morphology of the adhesive organs by light- and electron microscopy. We performed transcriptome sequencing and differential gene expression analysis to obtain tail-specific candidate transcripts. An *in situ* hybridization screen revealed the expression of these transcripts in the tail and allowed the identification of transcripts expressed in the adhesive organs. Functional analysis by RNA interference (RNAi) corroborated the involvement of several transcripts in the *M. ileanae* adhesion process. Multiple approaches confirmed the presence of adhesive proteins in the footprints. Sequencing of genomic DNA (gDNA) using Oxford Nanopore confirmed that two transcripts belonged to a larger, single adhesive protein and that repetitive sequence motifs were present. A better understanding of *M. ileanae* temporary adhesion will contribute to unravelling the cell biology and evolution of flatworm adhesive systems. Furthermore, the identification of new flatworm glue proteins can lead to the generation of a biomimetic glue with novel properties.

## 2. Results

### (a) Morphology of adhesive organs

**Figure 1.**
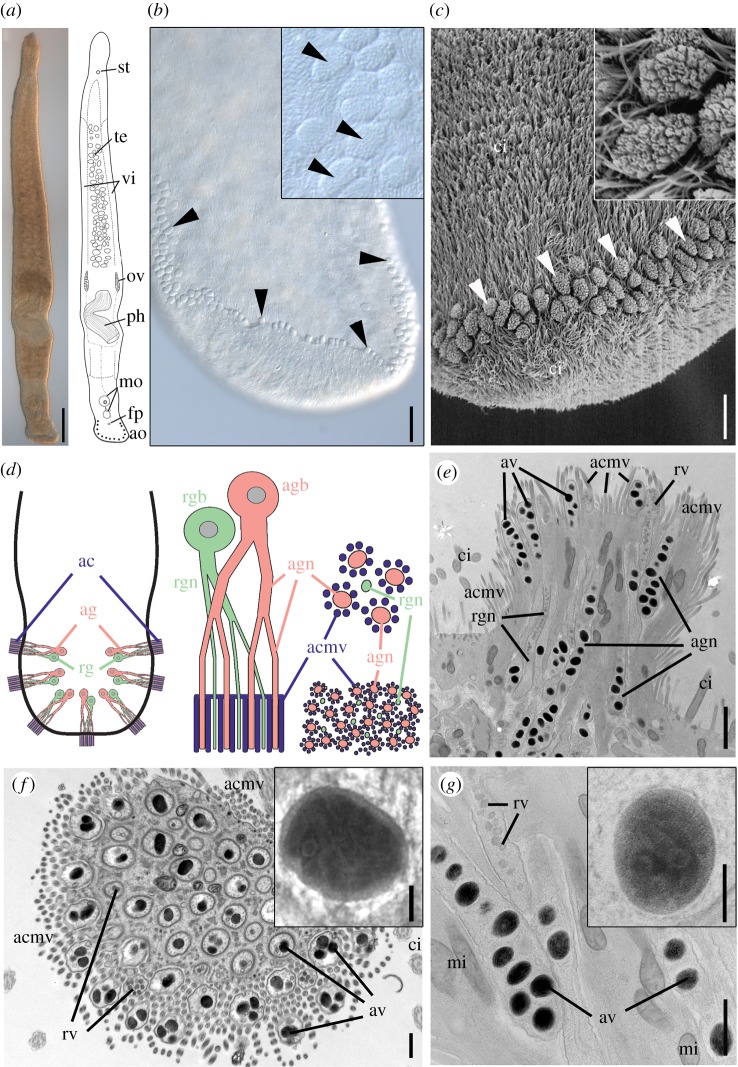
Morphology of *Minona ileanae* and the adhesive organs. (*a*) Differential interference contrast and schematic drawing. (*b*) Detail of the ventral side of the tail-plate (posterior-most tip slightly folded inside) with adhesive organs (arrowheads); detail of adhesive pads (inset). (*c*) Scanning electron microscopy of posterior tip of the tail with adhesive organs (arrowheads) and detail (inset). (*d*) Left: scheme of the organization of adhesive organs in the tail (seven organs shown); middle: scheme of a single adhesive organ with anchor cell (blue), adhesive gland cell (pink), and releasing gland cell (green); bottom right: scheme of a cross-section through the apical region of an adhesive organ (compare with electronic supplementary material, figure S1*c*,*d*); top right: detail of four distal ends of adhesive gland cell neck branches (pink) surrounded by microvilli of the anchor cell (blue) and one distal end of a releasing gland cell branch (green). (*e*–*g*) Transmission electron micrographs of adhesive organs: (*e*) longitudinal section from cryo-fixed sample and (*f*) cross-section through the organ's apical region (chemical fixation). Inset in (*f*) shows an adhesive vesicle with clear core–periphery zonation of the contents (lead staining). (*g*) Detail of adhesive and releasing gland cell necks and magnification of one adhesive vesicle with substructures (inset) (uranyl acetate/lead staining of cryo-fixed sample). ac, anchor cell; acmv, anchor cell microvilli; ag, adhesive gland; agn, adhesive gland neck; agb, adhesive gland cell body; ao, adhesive organs; av, adhesive vesicles; ci, cilia; fp, female pore; mi, mitochondria; mo, male organ; ov, ovary; ph, pharynx; rg, releasing gland; rgb, releasing gland body; rgn, releasing gland neck; rv, releasing vesicles; st, statocyst; te, testes; vi, vitellaria. Scale bars (*a*) 200 µm, (*b*) 25 µm, (*c*) 10 µm, (*e*) 1 µm, (*f*,*g*) 500 nm, inset (*f*,*g*) 100 nm.

We analysed the adhesive system of the proseriate flatworm *Minona ileanae* (figure 1*a*) [16]. *Minona ileanae* occurs in sand habitats of the intertidal zone. The animals grew up to 4 mm in length, and they moved actively in a snake-like manner (electronic supplementary material, movie M1). At the ventral end of the tail, up to 100 adhesive papillae—also called adhesive pads—were present in a horse-shoe-shaped formation (figure 1*b*). The cushion-shaped adhesive papillae occurred in two or three rows (figure 1*c*). Each adhesive papilla consisted of three cell types: a modified epidermal cell, called an anchor cell, which was penetrated by multiple branches of adhesive and releasing gland cell necks (figure 1*d*,*e*). The anchor cell lacked cilia but possessed actin-enforced elongated microvilli. These microvilli formed a collar around the distal ends of each adhesive gland cell opening but not around the ends of the releasing gland cell necks (figure 1*e*,*f*; electronic supplementary material, figure S1*a*–*d*). The ultrastructural characteristics of the adhesive vesicles were generally similar in chemically fixed or cryo-fixed specimens (see electronic supplementary material). The adhesive vesicles were ovoid, measuring 455 ± 62 nm × 213 ± 20 nm (*n* = 60) in chemically fixed samples and 270 ± 24 nm × 160 ± 17 nm (*n* = 44) in cryo-fixed samples. These size differences likely result from artefactual swelling of the vesicles during chemical fixation, as reported from other glands [17]. The vesicle contents showed a clear internal zonation with an electron-dense core and a moderately electron-lucent periphery (figure 1*f* inset) in lead-stained sections. Cryo-fixed vesicles post-stained with uranyl acetate plus lead showed further ultrastructural details (figure 1*g* inset). Their core versus periphery zonation was no longer visible, thus obviously masked by the additional staining step. Even more interesting was that linear substructures were consistently observed throughout the vesicle matrix, but were barely visible in chemically fixed samples. This still-unexplained feature has not yet been reported for other flatworm adhesive vesicles. The spherical releasing gland cell vesicles (figure 1*g*) appeared moderately electron-dense, with a diameter of 98 ± 8 nm (*n* = 75) in chemically fixed samples and 83 ± 10 nm (*n* = 45) in cryo-fixed samples. Both adhesive and releasing gland cells showed branching of the gland cell necks (electronic supplementary material, figure S1*e*,*f*). The nucleus of the adhesive gland cell was located below the epidermis and the body wall musculature (electronic supplementary material, figure S2). In proximity to the adhesive gland cell nucleus, the gland cell necks had a larger diameter (multiple adhesive vesicles per cross-section) compared with the diameter of the branched necks within the adhesive papilla (one or two vesicles). Similar to the adhesive gland cells, the cell nuclei of releasing gland cells were located below the body wall musculature (electronic supplementary material, figure S2). At the base of an adhesive papilla, the adhesive and releasing gland cell necks penetrated the anchor cell in bundles (electronic supplementary material, figure S3*a*). Nitrogen content was measured with electron energy loss spectroscopy (EELS) and visualized with electron spectroscopic imaging (ESI). The nitrogen content was high in adhesive vesicles but low in releasing vesicles (electronic supplementary material, figure S3*b*,*c*). In summary, the adhesive organs of *M. ileanae* possessed the cell types characteristic of flatworm adhesive organs.

### (b) Transcriptome and differential gene expression

We generated Illumina libraries from three biological replicates of adult worms of mixed age and performed Illumina paired-end 100 bp sequencing. A de novo transcriptome of *M. ileanae* was assembled using Trinity version v2.0.6. The final transcriptome assembly accounted for 264 995 transcripts, with an N50 of 1132 bp in length, and a GC–content of 36.4% (electronic supplementary material, table S1). CD-HIT clustering [18,19] was used to reduce the number of highly identical (98% identity) transcripts, resulting in a transcriptome that contained 231 117 transcripts. For the assessment of transcriptome completeness, busco software was applied [20,21]. The analysis resulted in the following busco numbers: 94.6% [*D*: 32.1%], *F*: 1.6%, *M*: 3.8%, *n*: 978. These data confirmed that the generated *M. ileanae* transcriptome was of high quality and can be used as a valuable source for downstream applications.

**Figure 2.**
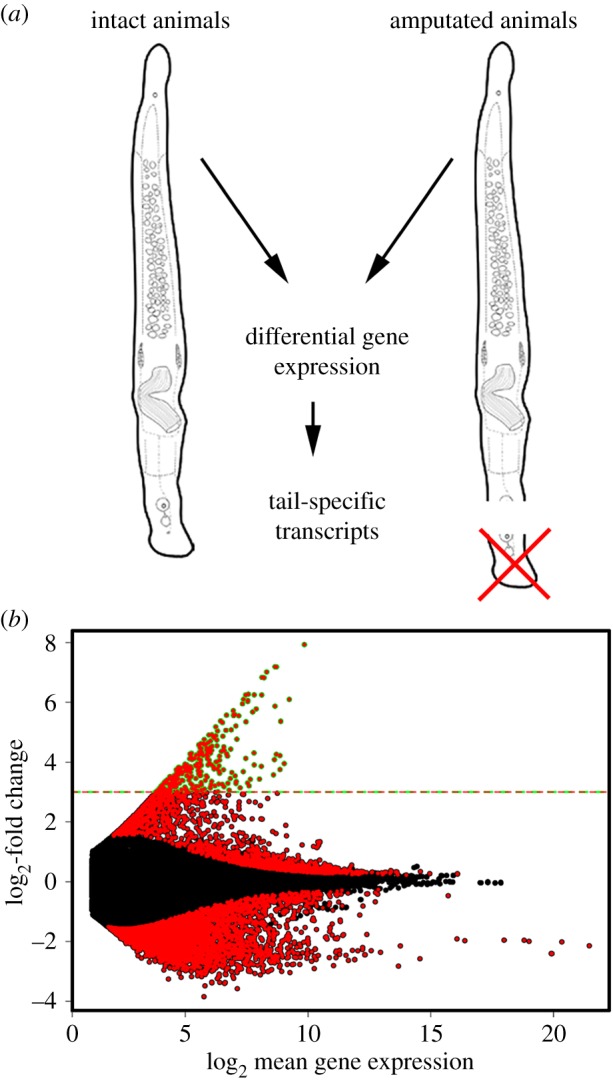
Transcriptome and differential gene expression. (*a*) Illustration of the strategy of the differential gene expression approach. (*b*) Differentially expressed transcripts with an adjusted *p*-value < 0.01 are indicated in red. Transcripts with an eightfold expression or higher (dashed line) were considered for further analyses.

For the identification of tail-specific transcripts, we performed differential RNA-seq (figure 2*a*). Illumina libraries from three biological replicates of intact animals and three biological replicates of tail-amputated worms were produced. Illumina 50 bp sequencing was performed. To determine differentially expressed transcripts in the tail, the reads from the intact worms and those from the amputated worms were mapped against the *M. ileanae* transcriptome. Differentially expressed transcripts were identified using the DESeq2 software package [22]. From all differentially expressed transcripts with an adjusted *p*-value < 0.01 (figure 2*b*), a list of 326 transcripts exhibited an eightfold higher expression in the tail (figure 2*b*: dashed line; electronic supplementary material, dataset S1).

### (c) *In situ* hybridization screen and characterization of adhesive organ-specific transcripts

We expected to localize adhesion-related gland cell candidates inside the tail-plate at the level of gland cell bodies, whereas anchor cell-specific transcripts should be located towards the periphery of the tail (figure 1*d*). To generate a highly tail-specific candidate list for the *in situ* hybridization screen, the following selection criteria were applied: (i) eightfold differential expression between intact and amputated animals, (ii) an average number 25+ mapped reads in intact animals for the respective transcript, (iii) selection of the longest isoform of similar transcripts, (iv) presence in footprints as identified by mass spectrometry, and (v) homology to *M. lignano* adhesion-related proteins. Fifty-two transcripts were selected for *in situ* hybridization (electronic supplementary material, dataset S1), and the respective expression pattern was determined.

Nine transcripts showed expression in the adhesive organs, with eight being expressed at the site of adhesive and releasing gland cell bodies (electronic supplementary material, figure S4*a*–*h*). Owing to the close vicinity of adhesive and releasing gland cell bodies we could not discriminate between these two cell types based on the expression data. Four transcripts were associated with female reproductive structures (electronic supplementary material, figure S5*a–d*), 34 transcripts were related to the male reproductive organs (electronic supplementary material, figures S5*e–u* and S6*a–q*), and three showed other expression patterns (electronic supplementary material, figure S6*r,t*). For the remaining five transcripts, no expression pattern could be obtained.

**Figure 3.**
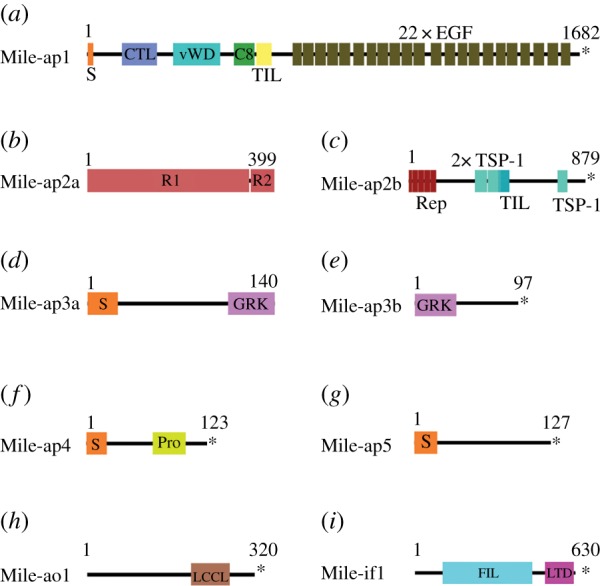
Conserved domain architecture of adhesion-related proteins. Abbreviations: C8, C8 domain; CTL, C-type lectin domain; EGF, epidermal growth factor-like domain; FIL, intermediate filament protein domain; GRK, glycine-arginine- and lysine-rich region; LTD, lamin tail domain; LCCL, LCCL domain; Pro, proline-rich region; R1(2), repeat unit 1(2); Rep, repeat region; S, signal peptide; TIL, trypsin inhibitor-like cysteine-rich domain; TSP-1, thrombospondin 1 domain; vWD, von Willebrand factor type D domain; * indicates a stop codon.

We determined the conserved protein domains for the transcripts expressed in the adhesive organs (figure 3). Mile-ap1 (*M. ileanae* adhesive protein 1) showed high similarity to the *M. lignano* adhesive protein 1 (Mlig-ap1) with respect to the presence of protein domains, such as a C-type lectin domain (CTL), a von Willebrand domain (vWD), a C8-domain (C8) and a trypsin inhibitor-like domain (TIL). In addition, multiple tandem repeats of calcium-dependent epidermal growth factor-like domains (EGF) were present, which are characteristic of the fibrillin protein family. A comparison of the Mile-ap1 amino acid sequence with the multi-domain core region of Mlig-ap1 showed a 39.92% sequence identity. However, lysine- and arginine-rich regions, characteristic for Mlig-ap1, were lacking in Mile-ap1. Mile-ap2a contained a protein repeat unit (see chapter ‘Oxford Nanopore gDNA sequencing’ for the presence of multiple copies of this repeat). The amino acid composition of the repeat region revealed numerous threonine residues, which could correspond to sites for glycosylation (electronic supplementary material, figure S7). Mile-ap2b had three thrombospondin 1 domains (TSP-1) and a TIL domain. Mile-ap3a and Mile-ap3b possessed multiple units of the peptide ‘glycine-arginine-lysine (GRK). Mile-ap4 contained a proline-rich region. Mile-ap5 had no known protein domain. Mile-ao1 (*M. ileanae* adhesive organ protein 1) possessed an LCCL domain (*Limulus* factor C, cochlear protein Coch-5b2 and late gestation lung protein Lgl1 domain). One transcript, *Mile-if1* (*M. ileanae* intermediate filament protein 1), with homology to the *M. lignano* anchor cell-specific intermediate filament protein Mlig-if1 [23], was expressed in anchor cells (electronic supplementary material, figure S4*i*,*i*′).

### (d) Functional analyses of adhesive organ-specific transcripts by RNA interference

**Figure 4.**
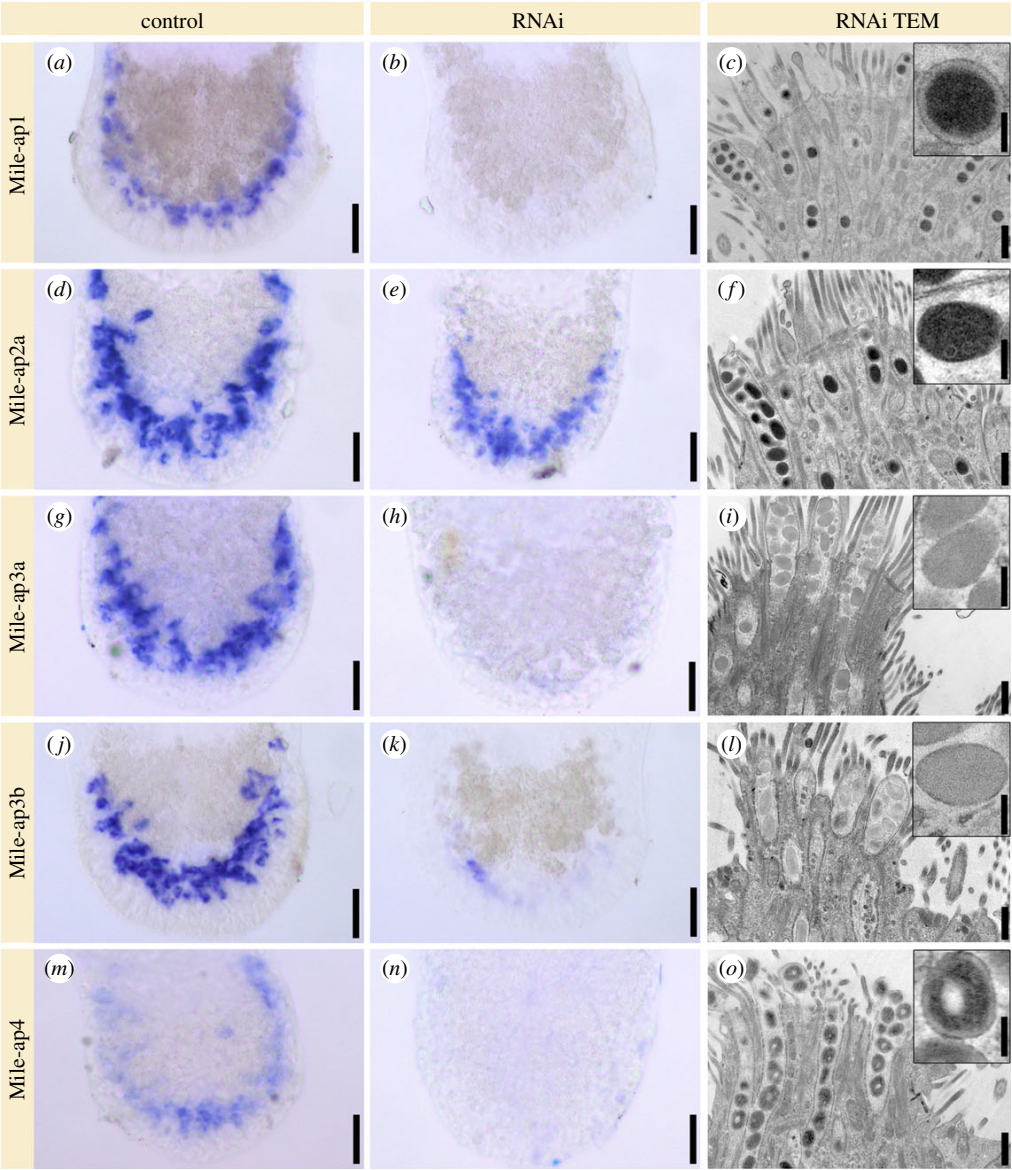
RNA interference (RNAi) of adhesion candidates. Expression of transcripts in the tails of *Minona ileanae*. *In situ* hybridization of control RNAi experiments (first column) and RNAi-treated animals (second column). Third column: transmission electron microscopic images of longitudinal sections of adhesive organs and details of an adhesive vesicle (insets) of RNAi-treated animals. All samples were chemically fixed and sections stained with lead. See text for details. Scale bars 25 µm (all control and RNAi *in situ* hybridizations), (*c*) 500 nm and 125 nm (inset) (same for all images of third column).

Next, we performed RNAi-mediated knock-down of all adhesion-related candidate transcripts (figure 4; electronic supplementary material, figure S8). We were not able to perform a standardized quantification of attachment capacity. However, using investigator-blinded observation by four independent researchers of controls or dsRNA-treated animals, a clear difference in the attachment capabilities of treated or untreated groups was identified. Unlike *Mile-ap5* and *Mile-ao1*, the transcripts *Mile-ap1-4* had a clearly reduced attachment phenotype. These observations were corroborated by *in situ* hybridization and ultrastructural alterations in the vesicle morphology of the adhesive gland cells. Treatment of animals with dsRNA of transcript *Mile-ap1* resulted in the abrogation of *Mile-ap1* mRNA (figure 4*a*,*b*) and induced spherical vesicles with a dense core and a very pronounced electron-lucent periphery (figure 4*c*, compared with wild-type animals, figure 1*f*: inset). RNAi of *Mile-ap2a* showed only partial reduction of the mRNA levels (figure 4*d*,*e*), but the adhesive vesicles lacked the electron-lucent periphery, while the substructures of the dense core were still visible (figure 4*f*). A similar phenotype was obvious for transcript *Mile-ap2b* (electronic supplementary material, figure S8*a*). Knock-down of either transcript, *Mile-ap3a* or *Mile-ap3b*, resulted in vesicles lacking the electron-dense core (figure 4*g*–*l*). Knock-down of *Mile-ap4* led to a conspicuous, concentric zonation of the vesicle contents with a lack of electron-dense material in the very core of the vesicles (figure 4*m–o*). *Mile-ap5* RNAi knock-down did not affect attachment: the adhesive vesicle ultrastructure resembled that of Mile-ap4 RNAi animals (electronic supplementary material, figure S8*b*). *Mile-ao1* did not show any effect on attachment capacity and showed no alteration of the vesicle ultrastructure (electronic supplementary material, figure S8*c*). *Mile-if1* RNAi resulted in a strongly reduced attachment capacity and a drastic ultrastructural change of the overall morphology of the adhesive pad (electronic supplementary material, figure S8*d*). As a control, luciferase dsRNA, a gene not present in *M. ileanae*, was used. No dsRNA mock effect regarding attachment capacity or the morphology of the adhesive organs and adhesive vesicles was observed (electronic supplementary material, figure S8*e*). Overall, RNAi knock-down of five transcripts resulted in an altered morphology of the adhesive vesicles and a reduced capacity of the animals to attach to the substrate.

### (e) Secretion of adhesive proteins

It is known from the flatworm *M. lignano* that adhesion proteins are secreted and remain on the substrate upon detachment of the animals. In order to study adhesive secretion in *M. ileanae*, we used antibody and lectin staining as well as mass spectrometry. We took advantage of an antibody generated against *M. lignano* adhesion protein Mlig-ap1 which shows a moderate similarity to Mile-ap3ab (see Material and methods (§4)) but can be used to stain *M. ileanae* footprints. Antibody staining revealed a horse-shoe-shaped imprint (electronic supplementary material, figure S9*a*) with marks of single adhesive pads. As the *M. lignano* adhesive protein Mlig-ap2 was shown to be glycosylated, we additionally tested 23 lectins (electronic supplementary material, table S2) for their potential to stain footprints. Eleven lectins showed specific labelling of *M. ileanae* footprints, indicating the presence of various sugar moieties (electronic supplementary material, table S2 and figure S9*b*–*l*). Transmission electron microscopy negative staining revealed a fibrous meshwork on the imprints (electronic supplementary material, figure S9*m*,*n*). The presence of secreted adhesive proteins was further studied using mass spectrometry. We analysed footprints of intact animals, footprints of tail-amputated animals, and footprints of amputated tails (for details, see electronic supplementary material and dataset S2). In the differential RNA-seq data, Mile-ap2a was not found to be tail-specific, but owing to its presence in the mass spectrometry samples, it was included in the *in situ* hybridization screen and in downstream analyses (electronic supplementary material, dataset S2). Overall, antibody staining and mass spectrometry confirmed the presence of adhesion-related proteins in *M. ileanae* footprints. Furthermore, lectin staining indicated the presence of glycoproteins in the adhesive secretions.

### (f) Oxford Nanopore gDNA sequencing

**Figure 5.**
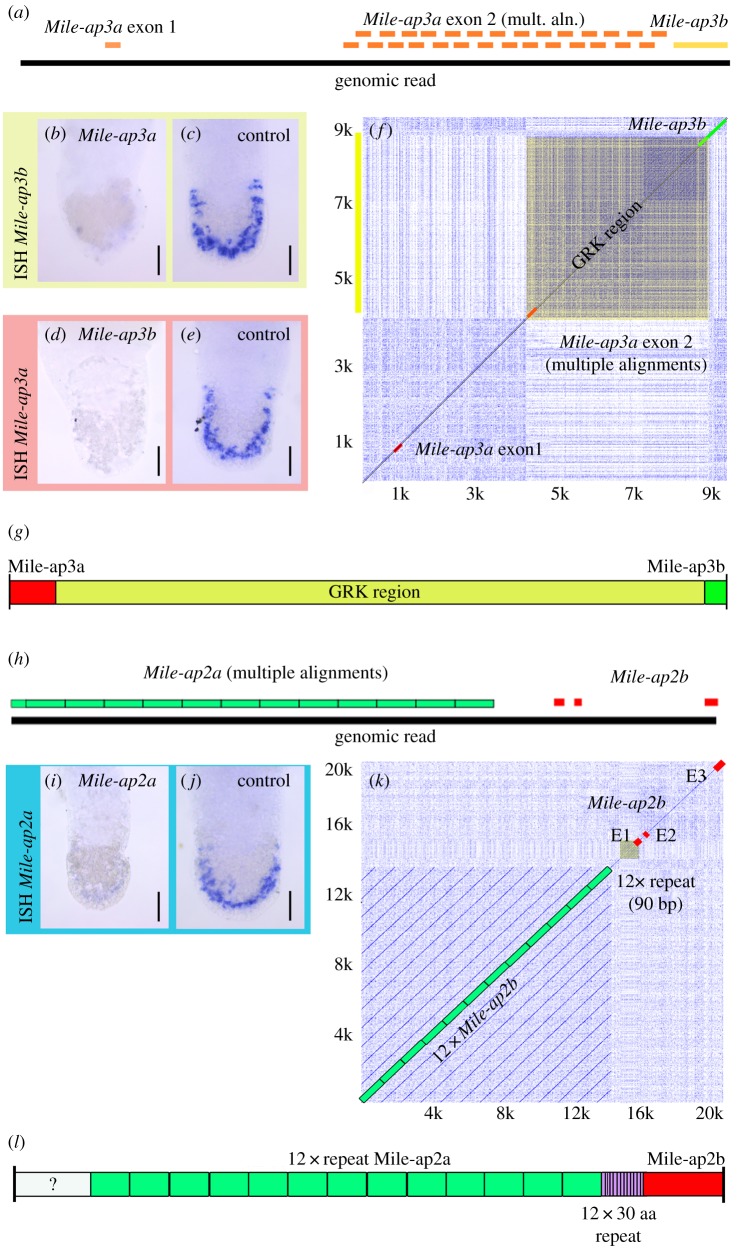
Oxford Nanopore sequencing of gDNA. (*a*) Mapping of *Mile-ap3a* and *Mile-ap3b* onto a single Oxford Nanopore read. (*b*,*c*) RNAi knock-down of *Mile-ap3a* and *in situ* hybridization with a probe of *Mile-ap3b*. mult. aln., multiple alignments. (*d*,*e*) RNAi knock-down of *Mile-ap3b* and *in situ* hybridization with a probe of *Mile-ap3a*. (*f*) Dot plot of a nine kb section of genomic read ‘27752bc6-c8c2-433f-8100-0d294e375058’. (*g*) Hypothetical adhesive protein containing Mile-ap3a and Mile-ap3b and an extensive GRK low complexity region. (*h*) Mapping of *Mile-ap2a* and *Mile-ap2b* onto a single Oxford Nanopore read. (*i*,*j*) RNAi knock-down of *Mile-ap2a* and *in situ* hybridization with a probe of *Mile-ap2b*. (*k*) Dot plot of a 20 kb section of genomic read ‘7de1b9a2-7996-4a1a-8b10-3f661449fd01’. (*l*) Hypothetical adhesive protein containing Mile-ap2a and Mile-ap2b and 12 repeats of Mile-ap2a. ISH, *in situ* hybridization.

It is known from flatworms and sea stars that adhesion-related proteins can be very long [9,12] and may contain extensive arrays of identical repeats. In conventional short-read transcriptomics, these long and repeat-rich transcripts do not assemble adequately, and successive identical repeats are not represented in the transcripts (electronic supplementary material, figure S10*a*). To cope with these issues, we performed a pilot study for a cost-effective method for addressing these problems. Using Oxford Nanopore sequencing of genomic DNA, we generated 6.25 Gb of sequencing data (776 590 reads) from a single gDNA library of *M. ileanae* (electronic supplementary material, figure S10*b*). The haploid genome size of *M. ileanae* was estimated by flow cytometry to be 571 Mbp (±2.7 s.d.) with *Drosophila* as an internal standard or 550 Mbp (±7.3 s.d.) with the rotifer *Brachionus asplanchnoidis* as internal standard (electronic supplementary material, figure S11). gDNA raw sequences were uploaded onto a custom BLAST server [24] for downstream analyses. BLAST searches against the Oxford Nanopore gDNA dataset allowed the identification of single gDNA reads containing exons of *Mile-ap3a* and *Mile-ap3b* (figure 5*a*). RNAi experiments of *Mile-ap3a* and *Mile-ap3b* corroborated that the two transcripts belonged to the same gene. Knock-down of transcript *Mile-ap3a* resulted in the abrogation of transcript *Mile-ap3b* (figure 5*b*,*c*). Likewise, knock-down of transcript *Mile-ap3b* resulted in the abrogation of transcript *Mile-ap3a* (figure 5*d*,*e*). In a dot plot matrix, a sequence written in the horizontal and vertical axes of the matrix is compared with itself. Multiple diagonal lines indicate repeat sequences, while box formation indicates low complexity regions. The dot plot of the gDNA sequence containing *Mile-ap3a* and *Mile-ap3b* revealed that a substantial low complexity glycine-arginine-lysine (GRK) region was present (figure 5*f*). From these observations, we can conclude that *Mile-ap3a* and *Mile-ap3b* transcripts were fragments of a larger hypothetical *M. ileanae* adhesion gene (figure 5*g*).

In a similar manner, transcripts *Mile-ap2a* and *Mile-ap2b* were also mapped to a single *M. ileanae* gDNA read (figure 5*h*). RNAi experiments of *Mile-ap2a* and *Mile-ap2b* confirmed that the two transcripts were fragments of the same gene (figure 5*i*,*j*). In addition, in dot plot analyses of the gDNA read, it became apparent that this gene contained at least 12 repeats of *Mile-ap2a* (figure 5*k*,*l*)*.* Therefore, these data confirm that Oxford Nanopore gDNA sequencing is a valuable tool for the analysis of large repeat-rich genes.

## 3. Discussion

Flatworms have evolved a remarkable temporary adhesion/release system to cope with the challenges of their marine or freshwater habitats. The animals can rapidly attach to and release from many surfaces. Recently, a model of how the animals achieve this task was proposed for the basal flatworm *Macrostomum lignano*. *Macrostomum lignano* possesses 130 adhesive organs with unbranched adhesive and releasing gland cells that penetrate the anchor cell in a pair-wise manner [23], an organization also seen in other species of Macrostomorpha [13,25]. For attachment in *M. lignano*, two adhesion proteins were secreted. Mlig-ap2 was considered to be the glue protein that interacts with the surface, whereas the second adhesive protein, Mlig-ap1, performed a cohesive function and mediated attachment between Mlig-ap2 and the microvilli of the *M. lignano* anchor cell. For detachment, it was proposed that the animals secreted a negatively charged molecule that interacted with the highly positively charged Mlig-ap1 to induce release of the animals. Both proteins, Mlig-ap1 and Mlig-ap2, remained as footprints on the substrate.

Here, for *Minona ileanae*, we discovered several modifications of this theme: (i) Adhesive organs were organized as adhesive pads, with adhesive and releasing gland cell necks exhibiting substantial branching and penetrating the anchor cell multiple times, resulting in adhesive papillae with an enlarged surface. (ii) Only adhesive gland cell necks were surrounded by the microvilli of the anchor cell. (iii) Three to five adhesive proteins were involved in *M. ileanae* attachment. (iv) The releasing molecule had to interact with the adhesive proteins from ‘outside’ the attachment points, i.e. the microvilli collar which surrounded the end of the adhesive gland cell necks. Based on these findings, we propose a model of *M. ileanae* attachment and release (electronic supplementary material, figure S12): When an adhesive pad comes into contact with the surface, the content of the adhesive vesicle—a mixture of adhesive proteins—is secreted. The protein mixture ensures attachment to the surface and cohesion to the tip of the microvilli of the anchor cell—the animal remains attached. For detachment, the releasing gland secretes an unknown component, which interferes with the attachment/cohesion proteins, resulting in the release of the animal (electronic supplementary material, figure S12).

The adhesive proteins of *M. ileanae* showed similarity to *M. lignano* adhesion proteins Mlig-ap1 and Mlig-ap2 and the cohesion protein ‘sea star footprint protein 1’ of *Asterias rubens*. It appears that different regions of the *M. lignano* adhesion proteins correspond to individual adhesive proteins of *M. ileanae*. For example, Mile-ap1 had very high resemblance to the core region of Mlig-ap1 (39.92% amino acid identity) with an identical order of protein domains (CTL, vWD, c8, TIL), while the KR-rich regions of Mlig-ap1 were part of the proposed Mile-ap3a/b protein (figure 5). In *M. lignano*, KR-rich regions of the large Mlig-ap1 protein were predicted to interact with a negatively charged releasing molecule and thereby induce detachment. Similar KR-rich regions were identified in *M. ileanae*, but in contrast to *M. lignano*, they were identified in the independent KR-rich protein Mlig-ap3a/b. In *Hydra*, the detachment was based on contractions of muscles in the basal disc, and therefore no releasing substance was required [26]. Mucus proteins with a protein domain architecture comparable with the ap1 adhesive proteins were present in *M. lignano* and *M. ileanae* (electronic supplementary material, figure S13*a*,*b*). Therefore, we hypothesize that KR-rich regions of an adhesive protein could have evolved from a mucus protein ancestor by enrichment of lysine and arginine within one or more regions of the mucus protein. Alternatively, KR-rich regions could have been added to a mucus protein by fusion with an independent KR-rich protein (electronic supplementary material, figure S13*c*). Trypsin inhibitor-like domain (TIL) and thrombospondin-like protein domains (TSP-1) of Mlig-ap2 were present in the proposed Mile-ap2a/b protein (figure 5). In addition, Mlig-ap2 was characterized by multiple repeats of unknown function. Likewise, the Mile-ap2a/b protein held 12 or more repeat units. The units were not conserved with respect to the primary amino acid sequence; nevertheless, the presence of protein repeats in two distant species could point to the relevance of repeat units for flatworm adhesion. Notably, the high threonine content of Mile-ap2a and Mlig-ap2 (repeat pattern B) could indicate *O*-linked glycosylation, since mucin-type glycoproteins are characterized by α-d-*N*-acetylgalactosamine attached to serine or threonine [27].

The assembly of transcripts containing multiple repeats or low complexity regions is usually incomplete in conventional short-read-based transcriptomes. Four adhesion-related transcripts of *M. ileanae* showed indications that they were fragments of larger genes (figure 5) but it was unclear whether these transcripts were linked. We used Oxford Nanopore gDNA sequencing because of the possibility of establishing this method as an in-house tool for a small laboratory and its lower costs compared with a PacBio approach. Oxford Nanopore sequencing of *M. ileanae* gDNA of a single flow cell resulted in roughly 10× the coverage of the *M. ileanae* genome. Indeed, this approach allowed us to link transcripts and identify repeats and low complexity GRK regions. Notably, the exact amino acid sequence could not be determined owing to the error rate of Oxford Nanopore single reads [28,29]. Current genome assemblers, such as Canu, recommend 20× to 60× coverage for proper genome assembly [30]; therefore, we did not proceed towards an assembly of the *M. ileanae* genome. However, the output of data per flow cell is likely to increase in the near future, enabling genome assembly from the output of a single flow cell. Nevertheless, this approach allowed us to identify single gDNA reads for Mile-ap3a and Mile-ap3b as well as for Mile-ap2a and Mile-ap2b.

Glycosylated proteins have been identified as glue components in various organisms, such as the sea star *A. rubens* [31], or the flatworms *M. lignano* [32] and *Schmidtea mediterranea* [33]. Here, we showed that lectin stainings of *M. ileanae* footprints were mainly specific against glucose/*N*-acetylglucosamine (PSA, GSL II) and galactose/*N*-acetylgalactosamine (Pha-E, Pha-L, PNA, GSL I, ECL, VVA, DBA), indicating that these sugars might play a role in bioadhesion (electronic supplementary material, figure S9). In *M. lignano* only PNA and RCA also showed staining of adhesive vesicles. A greater variety of lectin-positive stainings in adhesive cells were found in *S. mediterranea* (10) and *A. rubens* (11). Five *S. mediterranea* adhesion cell-positive stainings (PNA, GSL II, VVA, ECL and DBA) and six *A. rubens* adhesive disc-specific stainings (UEA I, SNA, PHA-e, PHA-L, GSL I and DBA) also labelled footprints of *M. ileanae*. Even though current knowledge of the function of these sugars in temporary adhesion is limited, it has previously been suggested that glycoconjugates contribute to adhesion and cohesion, possibly through electrostatic interaction and/or cross-linking [31,34,35].

We were not able to successfully perform whole-mount lectin and antibody stainings. Adhesive vesicles are often densely packed, and the epitopes of the proteins and glycoconjugates may not be accessible to lectins and antibodies. Similar to this, in *M. lignano*, antibodies against adhesive proteins only showed specific stainings in footprints [12]; and in *A. rubens*, the lectins SJA and PNA only labelled extracted footprint material under denaturing conditions [31].

The duo-gland adhesive system of flatworms shows a remarkable performance in their habitat: rapid attachment- and release in marine or freshwater environments and strong attachment under high flow conditions. To get closer to a fundamental understanding of flatworm adhesion biology, it is important to study both the topology of the adhesive organs and the proteins involved in attachment and release in diverse flatworm species. Owing to the lack of knowledge on the composition of the adhesives in other flatworm species, no correlations regarding adhesive organ morphology, adhesive composition or the habitat of animals could be made. A single *M. ileanae* adhesive pad contains several ends of the branched adhesive gland necks, which are surrounded by microvilli of the anchor cell, while the releasing gland ends are intercalated within the adhesive pad. Such a topology could serve as a model for technical solutions. The role of the individual *M. ileanae* adhesive proteins remains to be elucidated. The true glue protein could be used to design a *Minona*-biomimetic glue.

## 4. Material and methods

### (a) Animal cultures

*Minona ileanae* Curini-Galletti 1997 were kept in the dark at room temperature in glass petri dishes with artificial seawater. The Innsbruck culture was established from laboratory cultures from the Curini-Galletti lab at the University of Sassari, Italy. Their natural habitat is the superficial layer of marine sandy beaches. Animals were originally found in Elat, Israel [16] but they also occur in the Suez Canal, and they have colonized the Mediterranean Sea [36]. They were fed twice a week with freshwater amphipods *Gammarus fossarum*, Koch 1835. The amphipods were killed, chopped into pieces and rinsed in artificial seawater (ASW) for five minutes before being added to the animal cultures. After 3 h, the exoskeletons were removed and the ASW of the petri dishes was renewed.

### (b) Transcriptome assembly and differential gene expression analyses

Transcriptome assembly and differential gene expression were performed as described before [37]. For details, see the electronic supplementary material.

### (c) Whole-mount *in situ* hybridization (WISH) and RNA interference

Whole-mount *in situ* hybridization and RNAi were performed according to Lengerer *et al*. [23] with minor modifications. For details, see the electronic supplementary material.

### (d) Mass spectrometry sample preparation and data analysis

Mass spectrometry was performed as described before [12]. For details, see the electronic supplementary material.

### (e) Antibody labelling of footprints

Antibody labelling was performed according to Wunderer *et al*. [12]. For footprint labelling, the polyclonal antibody AP1_R2 was used (the antibody was generated against the peptide SRKPRRKNRKSRKP of *Macrostomum lignano*). Potential binding sites could be found in Mile-ap3a/b with six out of 14 amino acids similarity to the *Macrostomum* peptide. This antibody did successfully stain the *M. ileanae* footprints but did not stain the adhesive gland cells in whole-mount animals.

### (f) Transmission electron microscopy (including element analysis); scanning electron microscopy

Electron microscopy was carried out according to Wunderer *et al*. [12], except that scanning electron microscopy was performed with a Zeiss DSM950 SEM (Zeiss, Germany) and images were taken with a Pentax digital camera and the PK Tether v. 0.7.0 free software. For details, see the electronic supplementary material.

### (g) Adhesion and releasing test assays

Embryo dishes with RNAi-treated animals were observed by four individual researchers and evaluated for their ability to adhere by creating a water flow by pipetting or by shaking the embryo dish.

### (h) High molecular weight DNA isolation

Prior to DNA isolation 2 × 200 adults of *M. ileanae* were treated with *N*-acetyl-l-cysteine mucus stripping solution for 15 min on a rotator. High molecular weight genomic DNA was obtained by the use of the MagAttract HMW DNA Kit (Qiagen, Germany) using the manufacturer's protocol. DNA was eluted in 100 µl distilled pure water. High molecular weight DNA solution of both isolations was pooled and concentrated by Speedvac to a volume of 45 µl. Qubit measurement (Invitrogen, USA) showed a total DNA concentration of 61.6 ng µl^−1^.

### (i) Oxford Nanopore sequencing

Low coverage Oxford Nanopore gDNA sequencing was performed on an Mk1B MinION System using LSK-108 sequencing chemistry (Oxford Nanopore Technologies (ONT), Oxford, UK) according to the manufacturer's protocol with minor modifications. For details, see the electronic supplementary material.

## Supplementary Material

Supplementary Figures, Tables, and Materials and Methods

## Supplementary Material

Supplementary data S1 on gene selection for expression screening and differential RNA-seq

## Supplementary Material

Datasheets on Mass Spectrometry

## Supplementary Material

Movie of Minona ileanae

## Data accessibility

The gene sequences reported in this paper have been deposited in the GenBank database (Mile-ap1, MK854810; Mile-ap2a, MK854811; Mile-ap2b, MK854812; Mile-ap3a, MK854813; Mile-ap3b, MK854814; Mile-ap4, MK854815; Mile-ap5, MK854816; Mile-ao1, MK854817; Mile-if1, MK854818). The transcriptome and RNAseq data have been deposited with links to BioProject accession number PRJNA535405 in the NCBI BioProject database (https://www.ncbi.nlm.nih.gov/bioproject/). The mass spectrometry proteomics data have been deposited to the ProteomeXchange Consortium via the PRIDE partner repository, https://www.ebi.ac.uk/pride/ (dataset identifier PXD013631).
