# Supplementary material for: Temporary adhesion of the proseriate flatworm Minona ileanae
Source: Philos Trans R Soc Lond B Biol Sci. 2019 Sep 9;374(1784):20190194. doi: 10.1098/rstb.2019.0194 (PMC6745481; doi:10.1098/rstb.2019.0194)
Supplement: Supplementary Figures, Tables, and Materials and Methods [file rstb20190194supp1.docx]

**Supplementary Electronic Material**

**Temporary adhesion of the proseriate flatworm *Minona ileanae***

Robert Pjeta^1^, Julia Wunderer^1^, Philip Bertemes^1^, Teresa Hofer^1^, Willi Salvenmoser^1^, Birgit Lengerer^2^, Stefan Coassin^3^, Gertraud Erhart^3^, Christian Beisel^4^, Daniel Sobral^5^, Leopold Kremser^6^, Herbert Lindner^6^, Marco Curini-Galletti^7^, Claus-Peter Stelzer^8^, Michael W. Hess^9^, Peter Ladurner^1*^

^1^Institute of Zoology and Center of Molecular Biosciences Innsbruck, University of Innsbruck, 6020 Innsbruck, Austria

^2^Biology of Marine Organisms and Biomimetics, Research Institute for Biosciences, University of Mons, 7000 Mons, Belgium

^3^Division of Genetic Epidemiology, Department of Medical Genetics, Molecular and Clinical Pharmacology, Medical University of Innsbruck, Innsbruck, Austria.

^4^Department of Biosystems Science and Engineering, ETH Zürich, Basel, Switzerland

^5^Instituto Gulbenkian de Ciência, Oeiras, Portugal.

^6^Division of Clinical Biochemistry, Biocenter, Innsbruck Medical University, 6020 Innsbruck, Austria.

^7^Dipartimento di Medicina Veterinaria, Università di Sassari, 07100 Sassari, Italy.

^8^Research Institute for Limnology, University of Innsbruck, Mondsee, Austria

^9^Division of Histology and Embryology, Innsbruck Medical University, 6020 Innsbruck, Austria

*corresponding author

**Supplementary Figures S1-S13:**

**Supplementary Tables S1 and S2**

**Supplementary Materials and Methods**


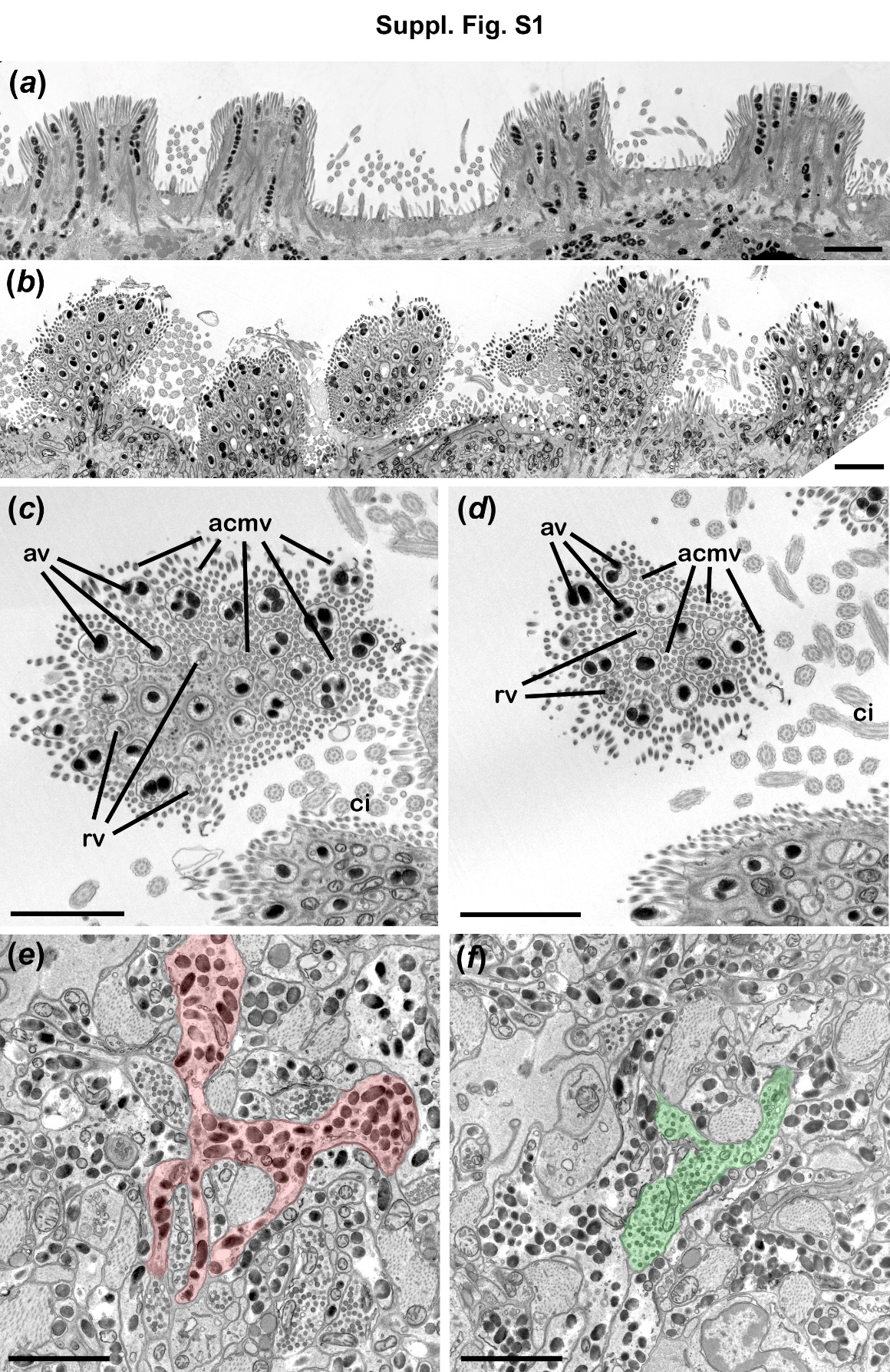


Supplementary figure S1. (*a*) Longitudinal section through four adhesive organs (*b*) oblique longitudinal section through five adhesive organs. (*c, d*) cross section through the apical region (*c*) and apical end (*d*) of an adhesive organ. (*e, f*) branching of an adhesive gland cell neck (*e*) and branching of a releasing gland cell neck (*f*). acmv anchor cell microvilli, av adhesive vesicles, ci cilia, rv releasing vesicles. Scale bars 2 µm.


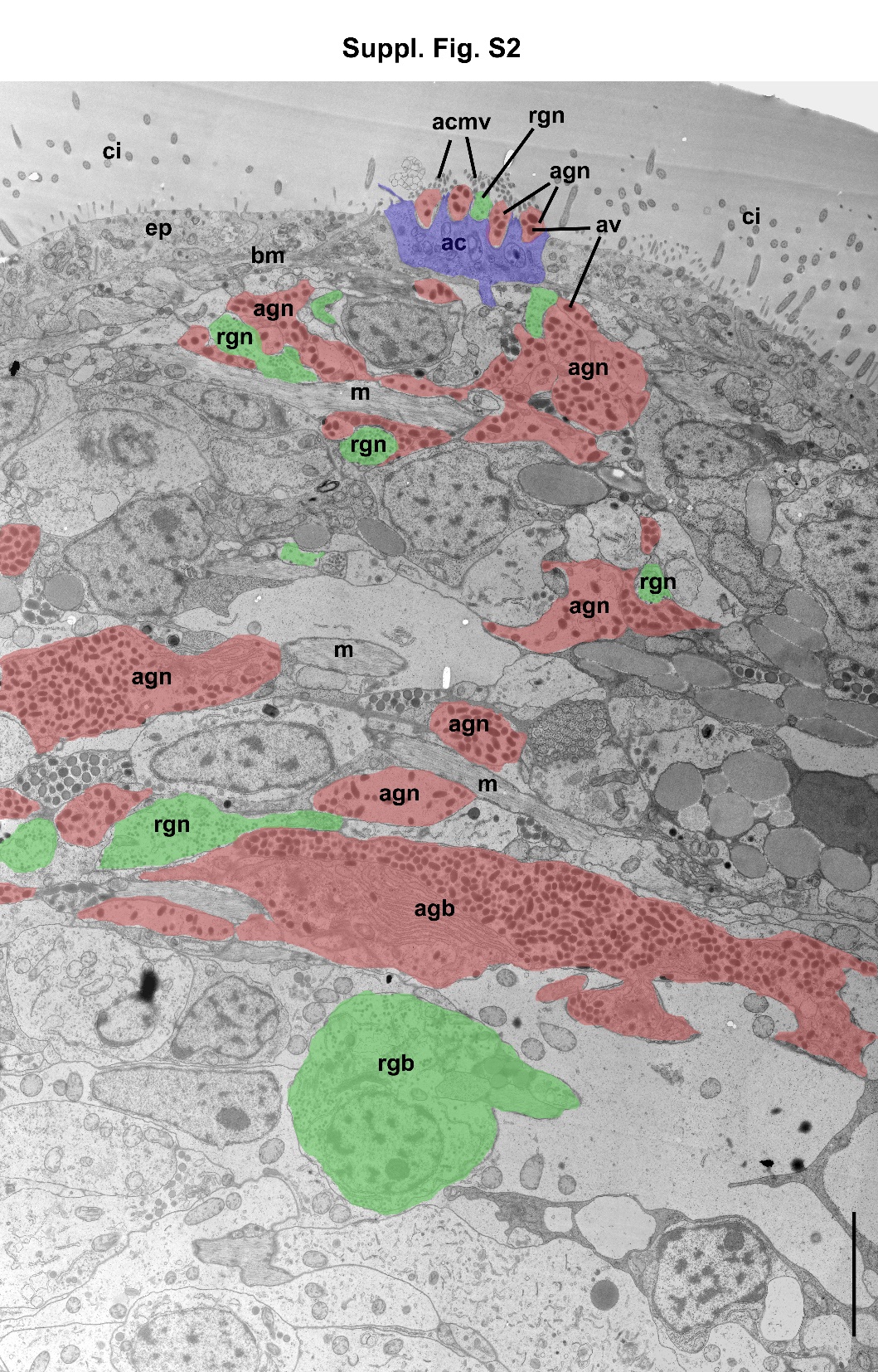


Supplementary figure S2. Cross section through the lateral region of the tail plate. Note the large diameter of adhesive- and releasing gland cell necks close to the cell bodies compared to the diameter at the papilla. ac anchor cell, acmv anchor cell microvilli, agb adhesive gland cell body, agn adhesive gland cell neck, av adhesive vesicles, bm body wall musculature, ci cilia, ep epidermis, m circular muscle, rgb releasing gland body, rgn releasing gland neck. Scale bar 4µm.


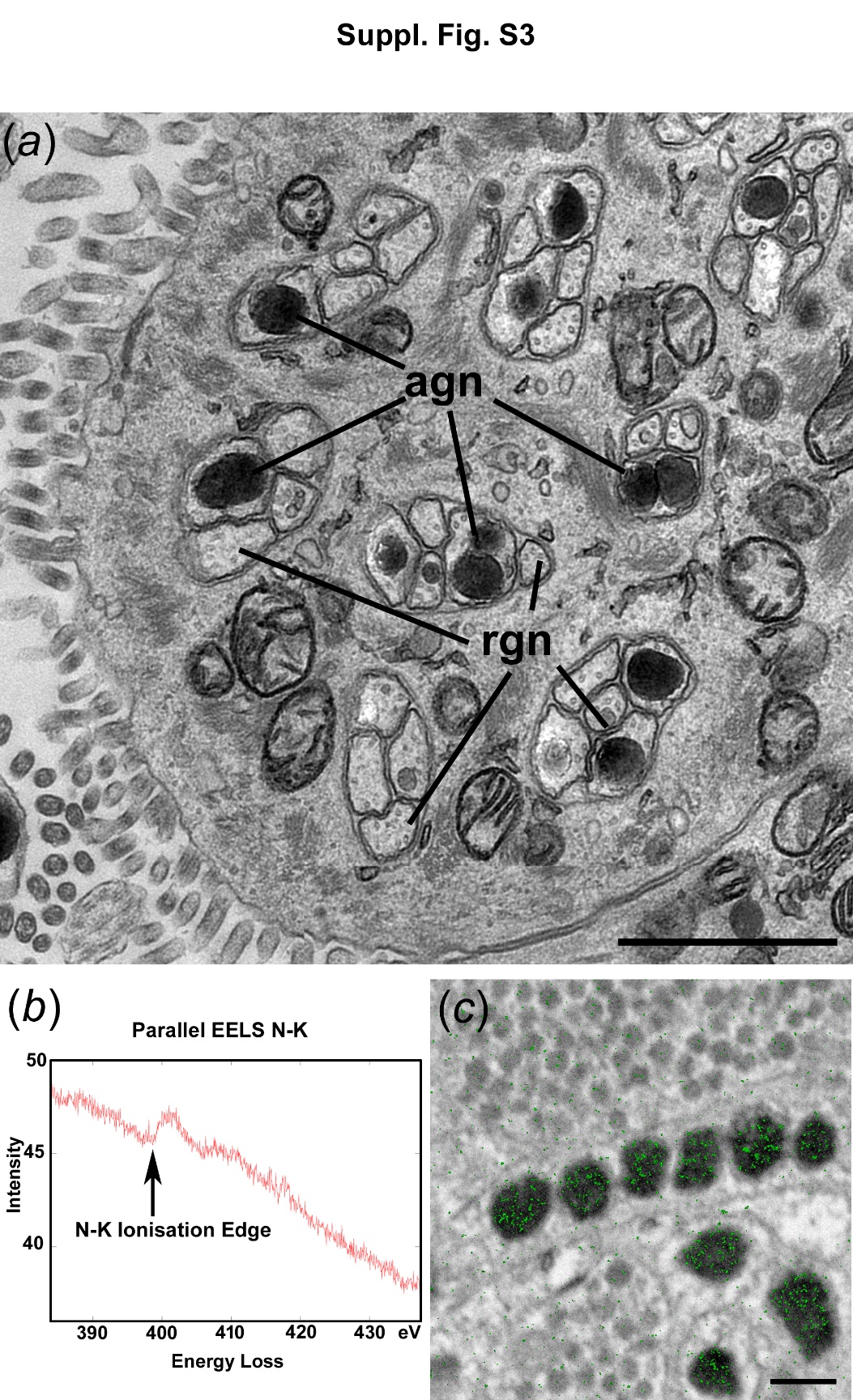


Supplementary figure S3. (*a*) Cross section at the base of an adhesive papilla. Note adhesive and releasing gland necks in bundles. (*b*) Electron Energy Loss Spectroscopy (EELS) of nitrogen of adhesive- and releasing vesicles showing the typical N-K Ionization Edge at 397 eV. (*c*) Electron Spectroscopic imaging (ESI) showing nitrogen enrichment (false colored in green) in adhesive vesicles. Scale bars (*a*), (*c*) 200 nm.


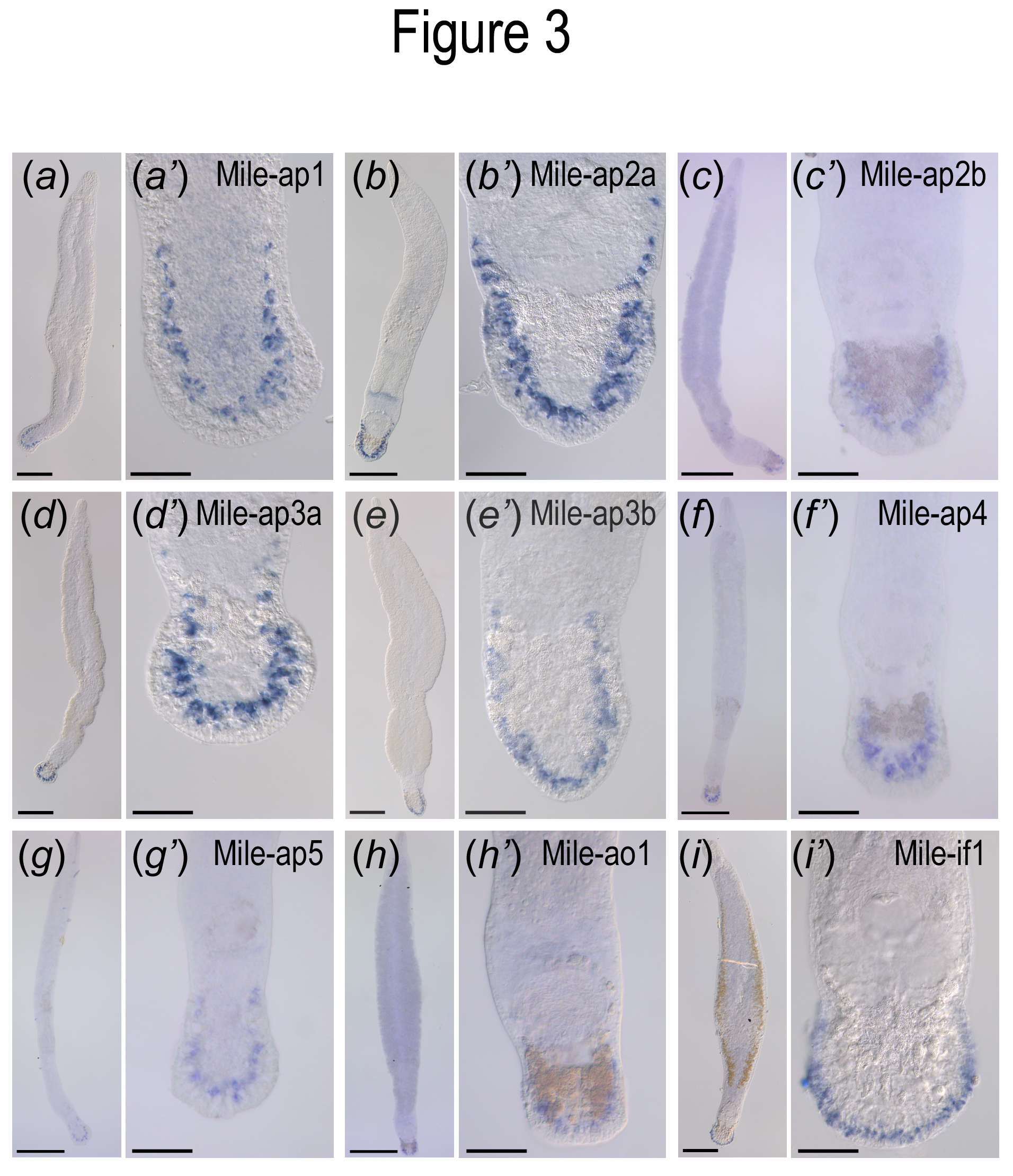


Supplementary figure S4. Expression of transcripts with overviews of whole animals and details of the tails. Scale bars 200 µm (overview panels), 50 µm (tail plate panels).


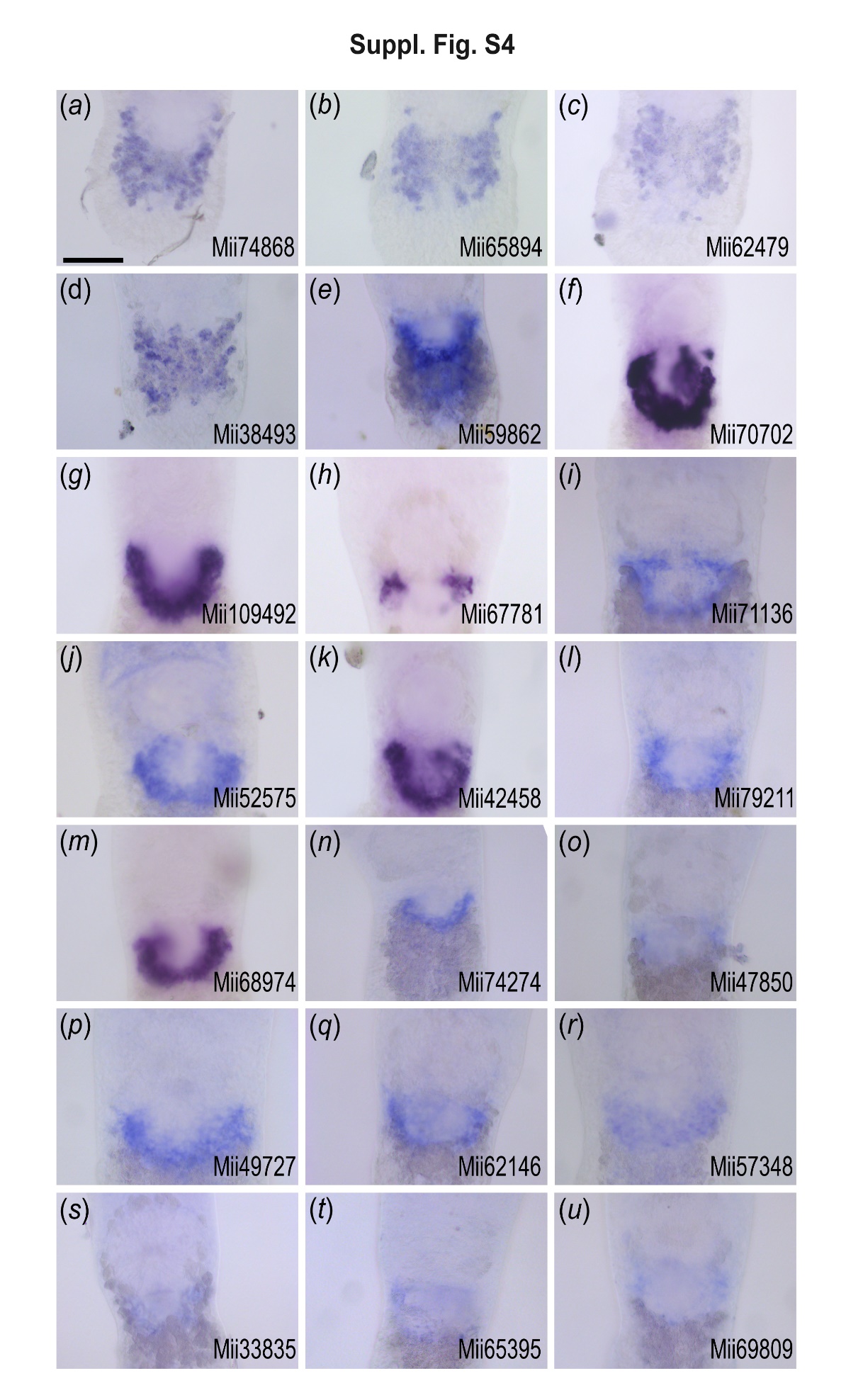


Supplementary figure S5. Expression of transcripts in *M. ileanae*. (a)-(d) Expression in the cement glands. Expression in (*e*)-(*u*) is associated to cells of the male copulatory system. Scale bar 50 µm.


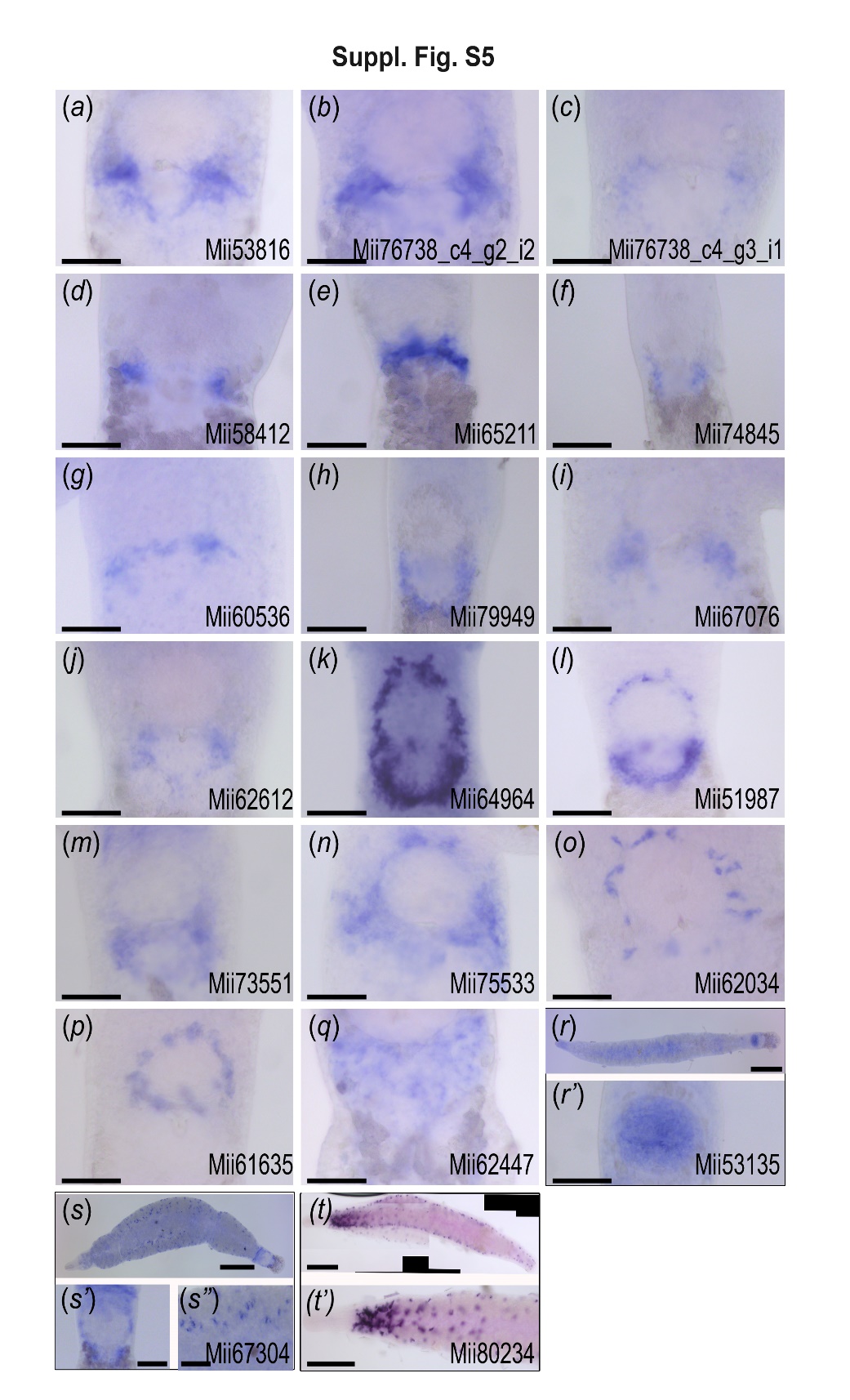


Supplementary figure S6. Expression of transcripts in *M. ileanae*. (*a*)-(*q*): Gene expression of male copulatory system related transcripts. (*r*) Expression in multiple tissues. Strongest signal can be seen in the copulatory bulb (*s*), (*t*) glands of the body wall. Scale bars (*a*)-(*q*) (*r’*) (*s’*) (*s’’*) (*t’*) 50 µm; (*r*) 200 µm; (*s*) 500 µm; (*t*) 100 µm
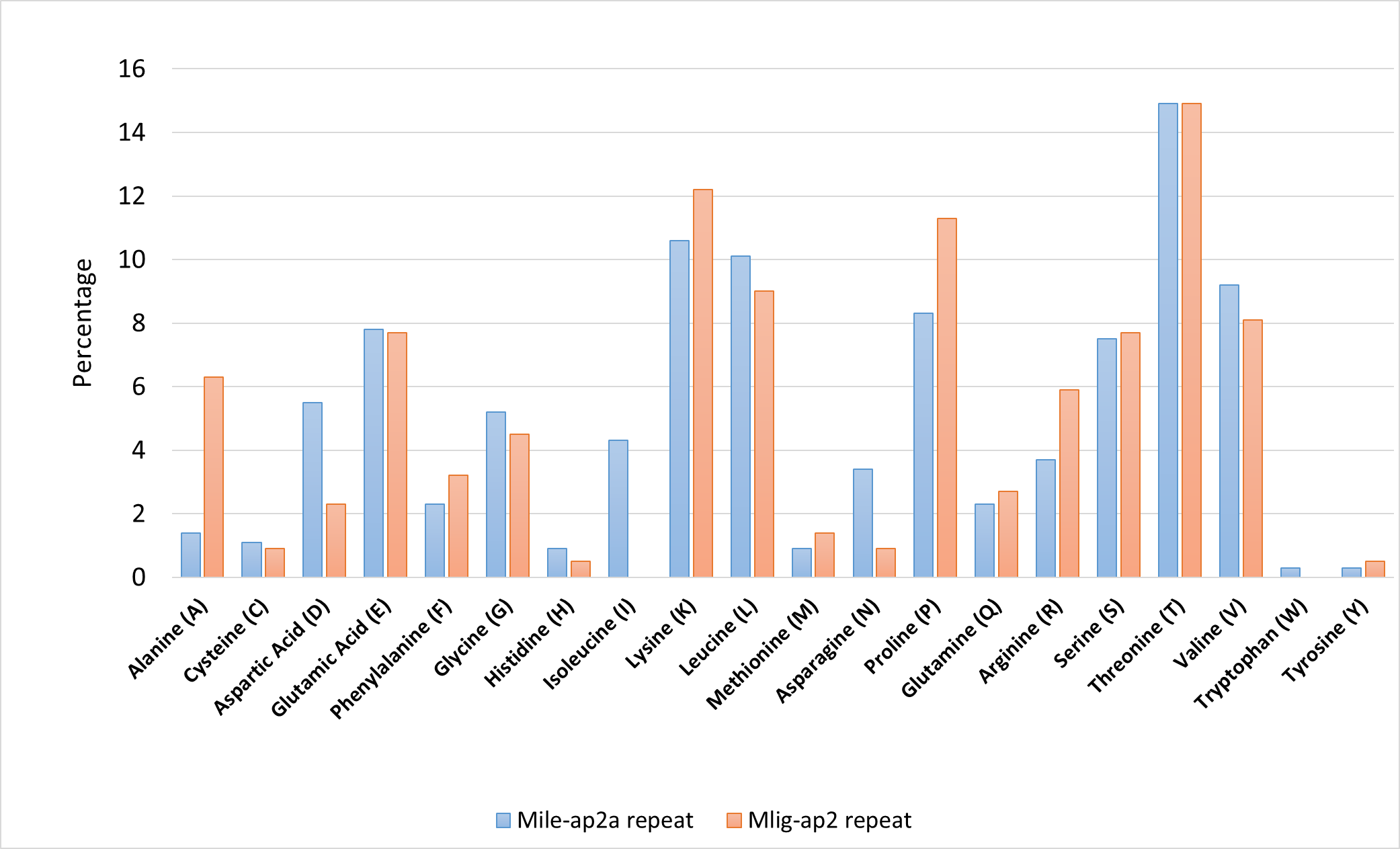


Supplementary figure S7. Amino acid composition of one Mile-ap2a repeat compared with one *M. lignano* Mlig-ap2 repeat.


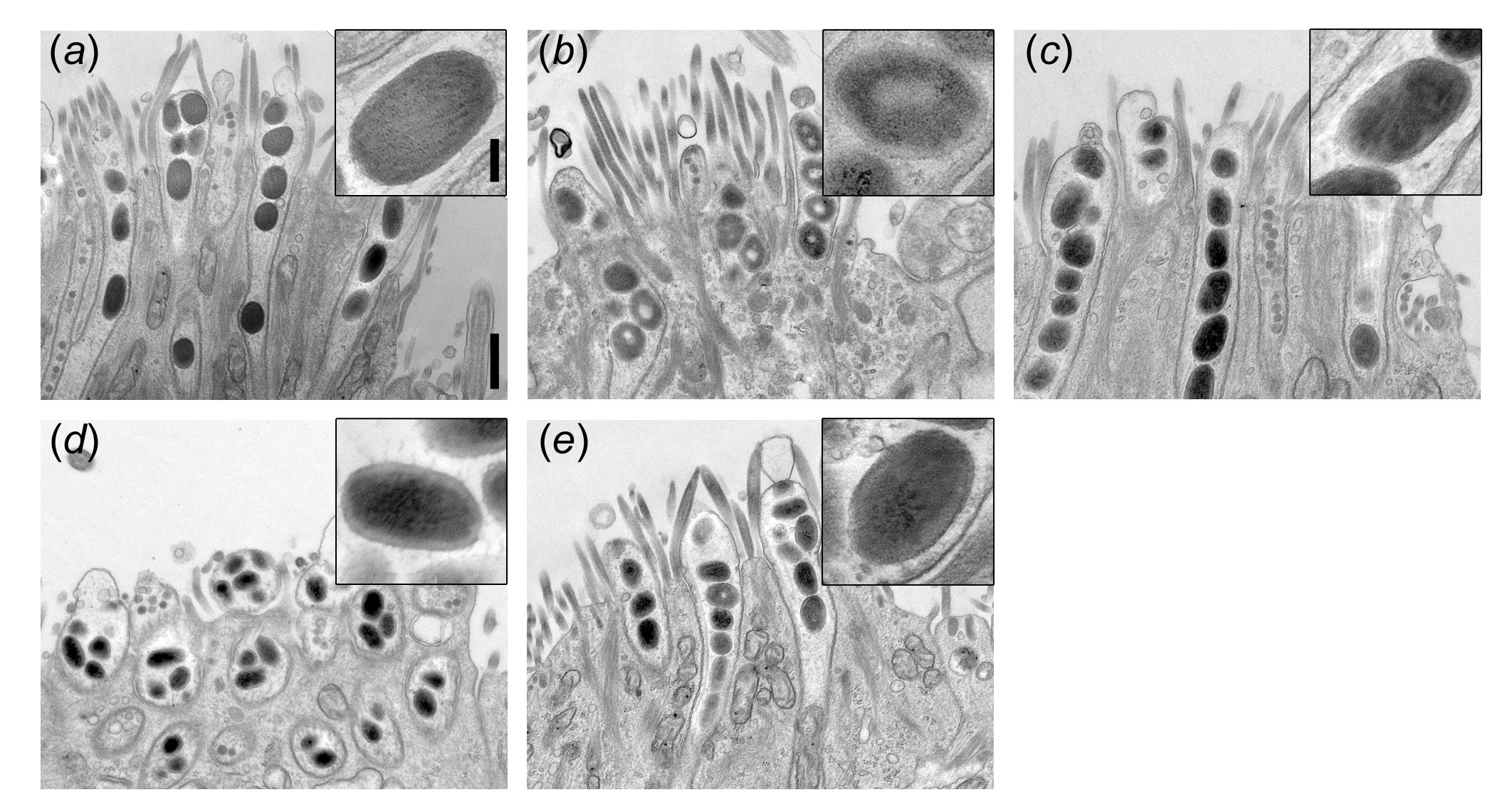


Supplementary figure S8. Transmission electron microscopic images of longitudinal sections of adhesive organs and details of an adhesive vesicle (insets) of RNAi treated animals. (*a*) *Mile-ap2b* (*b*) *Mile-AP5* (*c*) *Mile-ao1* (*d*) *Mile-if1* (*e*) *Luciferase* dsRNA treated. All samples chemically fixed and sections stained with lead. Scale bars (*a-e*) 500 nm (for all overview panels), 100 nm (for all insets).


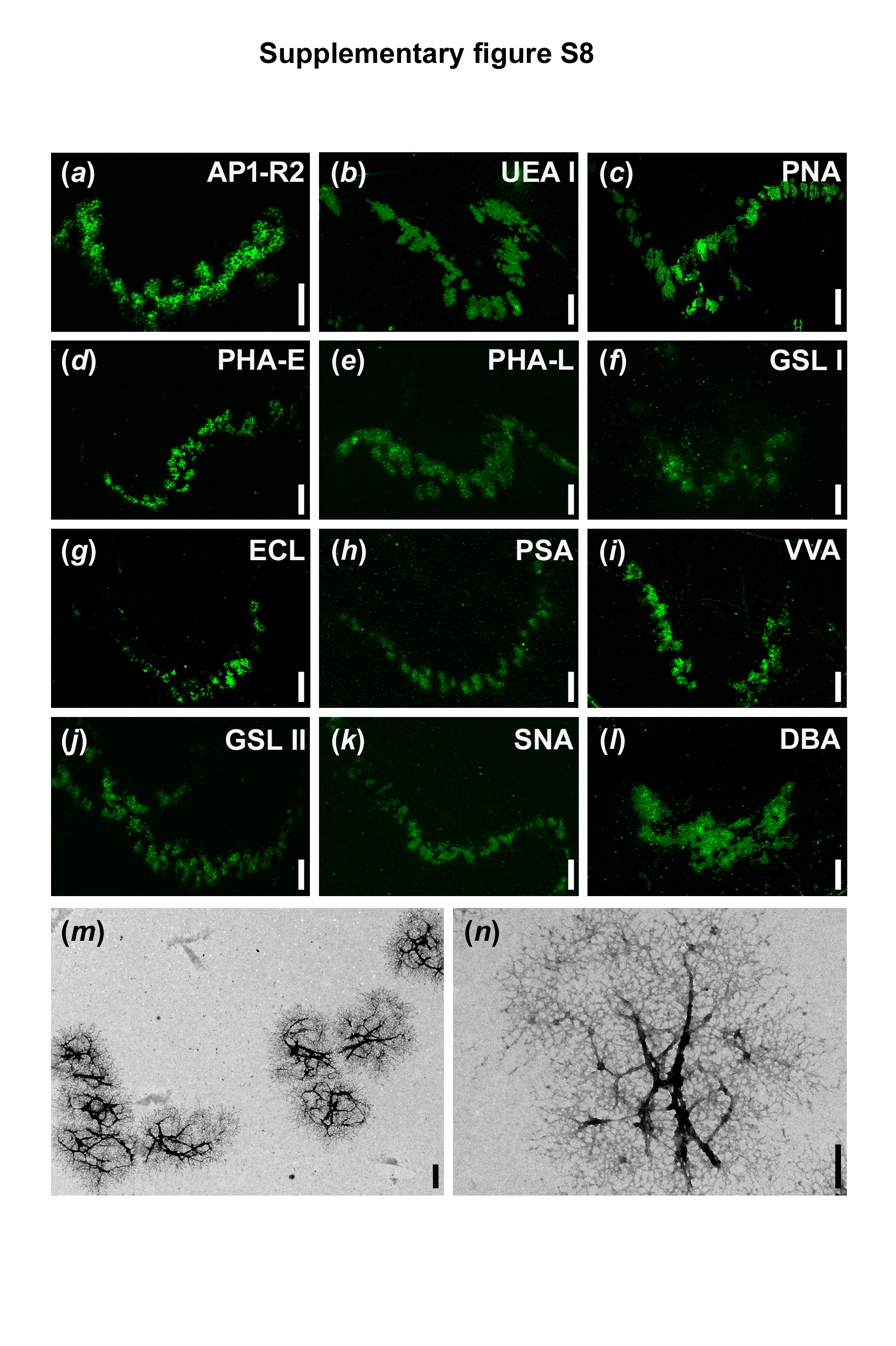


Supplementary figure S9. Antibody- and Lectin staining of footprints. (*a*) Footprint staining using the adhesion-protein specific *Macrostomum lignano* antibody AP1-R2. (*b*-*l*) Lectin staining of footprints. (*m, n*) Transmission electron microscopic negative staining of footprints. Imprints of two adhesive pads (*m*) and detail of an imprint (*n*). Scale bars (*a-l*) 20 µm, (*m*) 500 nm, (*n*) 250 nm.


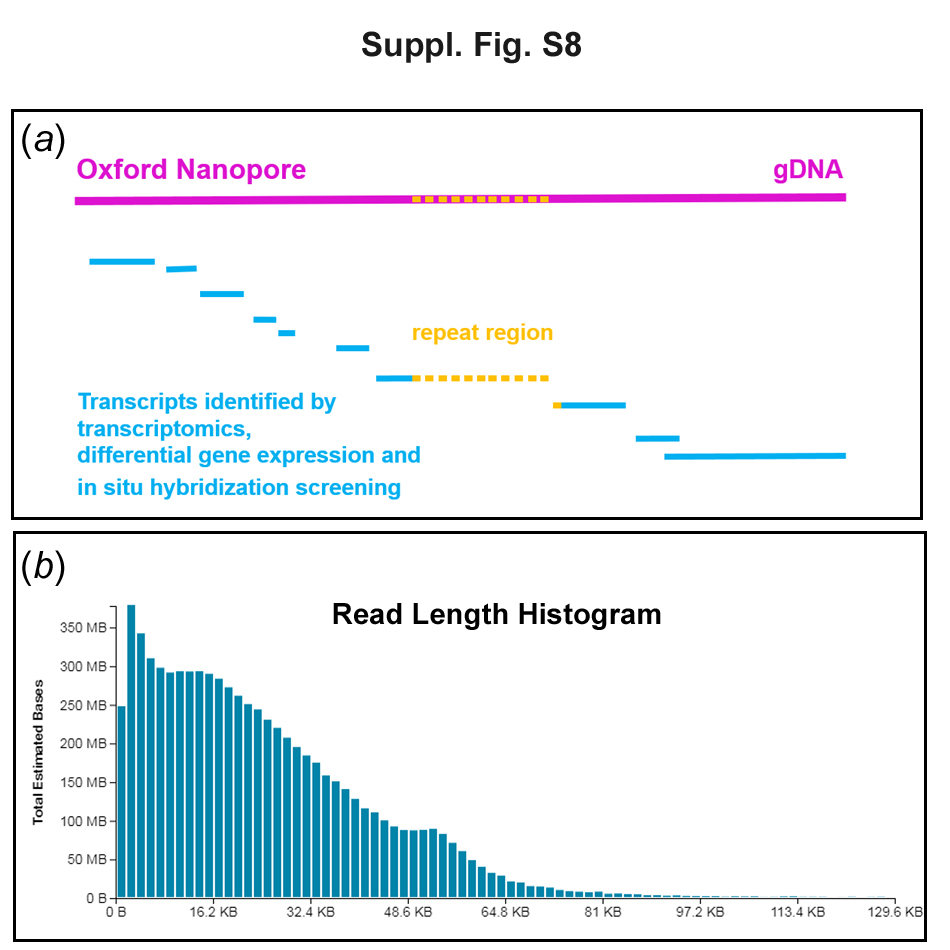


Supplementary figure S10. (*a*) Schematic drawing of a single Oxford Nanopore gDNA read (pink) and transcripts which were independent in a transcriptome (blue) but map to a long Oxford Nanopore read. The scheme illustrates that it is possible to link independent transcripts using long read technology. (*b*) Distribution of read length obtained from a single MinIon flow cell of *M. ileanae* high molecular weight gDNA.


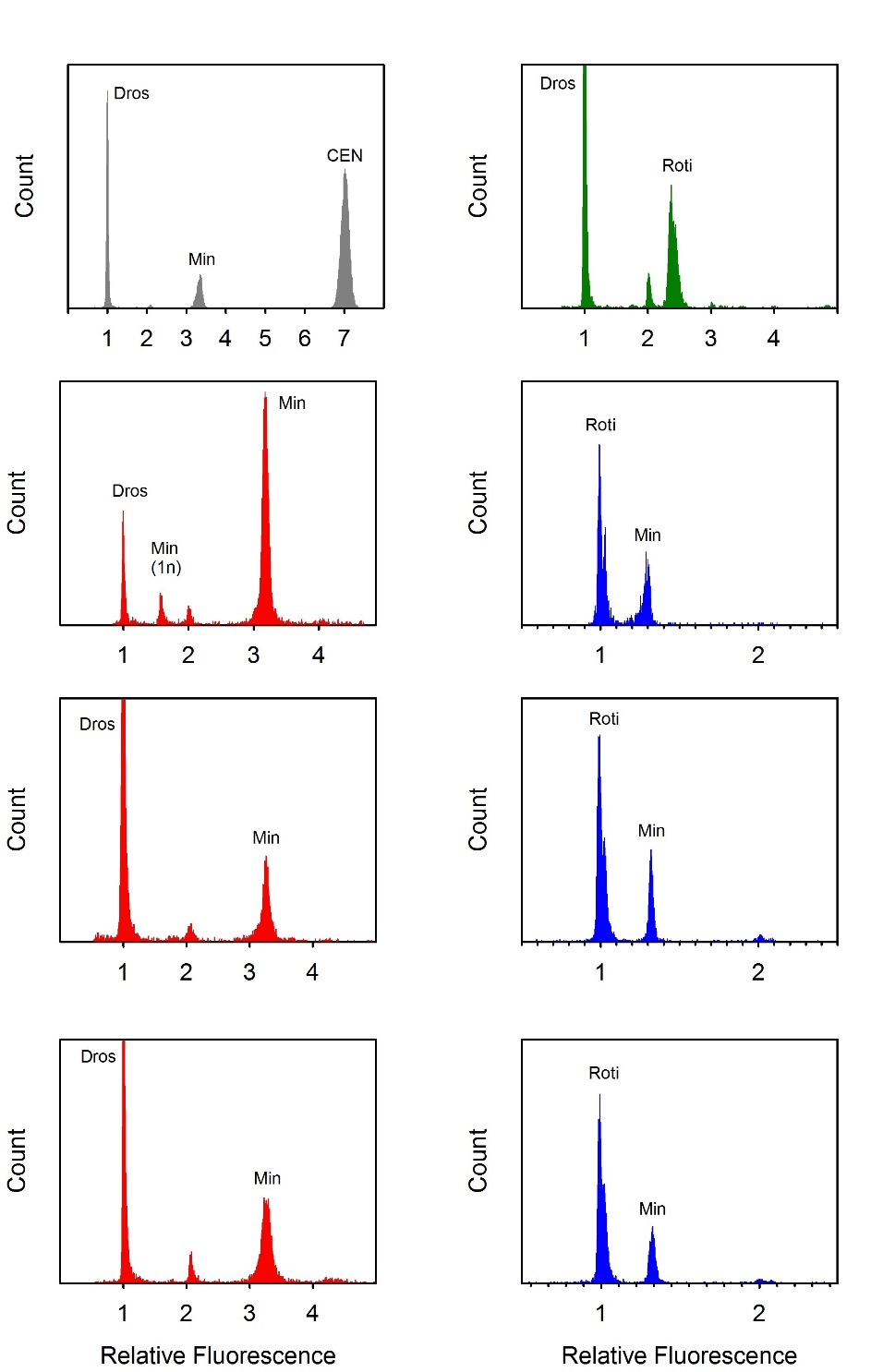


Supplementary figure S11. Flow-cytometric measurements of genome size in *Minona ileanae*. Histograms are based on the YL2-A fluorescence signals (detection bandwidth 590–650 nm). We rescaled the X-axis, so that the fluorescence values of *Minona* (Min) nuclei are displayed as multiples of the fluorescence of diploid *Drosophila* (Dros), or rotifer (Roti) nuclei. Grey: Sample combining two reference genomes (*Drosophila*; Chicken, CEN) and *Minona*; Red: Samples with *Drosophila* as internal standard; Green: Sample with Drosophila and Rotifer (*Brachionus asplanchnoidis*). Blue: Samples with *B. asplanchnoidis* as internal standard. Genome size estimated with *Drosophila* as internal standard was 571 Mbp (± 2.7 sd). Genome size estimated with *B. asplanchnoidis* as internal standard was 550 Mbp (± 7.3 sd).


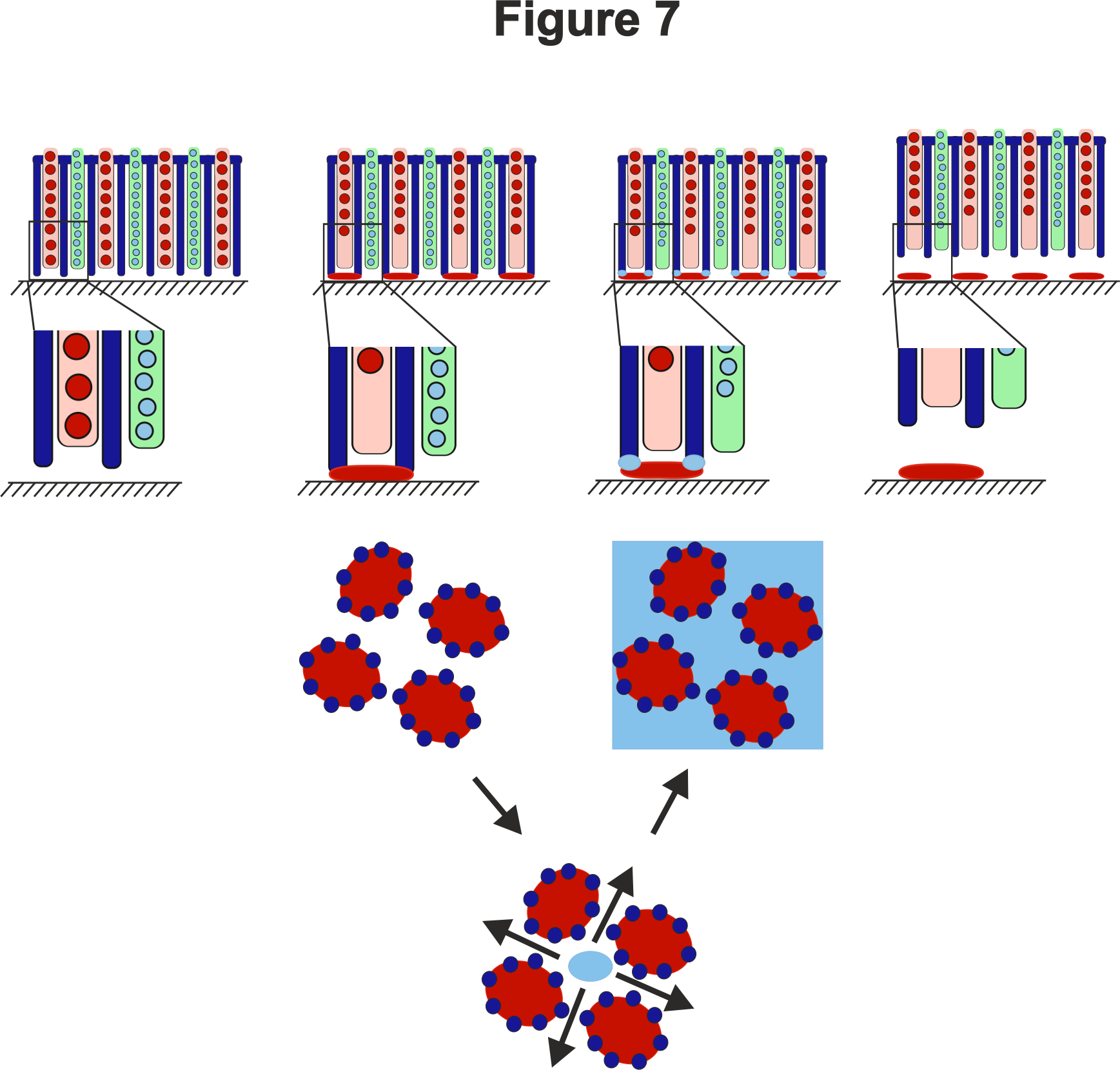


Supplementary figure S12. Model for adhesion and release in *Minona ileanae*. During attachment a protein mixture (red) was secreted from the adhesive gland cell (pink), which attached the tip of the microvilli (dark blue) to the surface. Upon release, an unknown releasing substance (light blue) was secreted by the releasing gland cell and interfered with the adhesion and cohesion proteins of the mixture. Consequently, the animal was released from the surface and a footprint of the adhesive protein mixture was left behind.


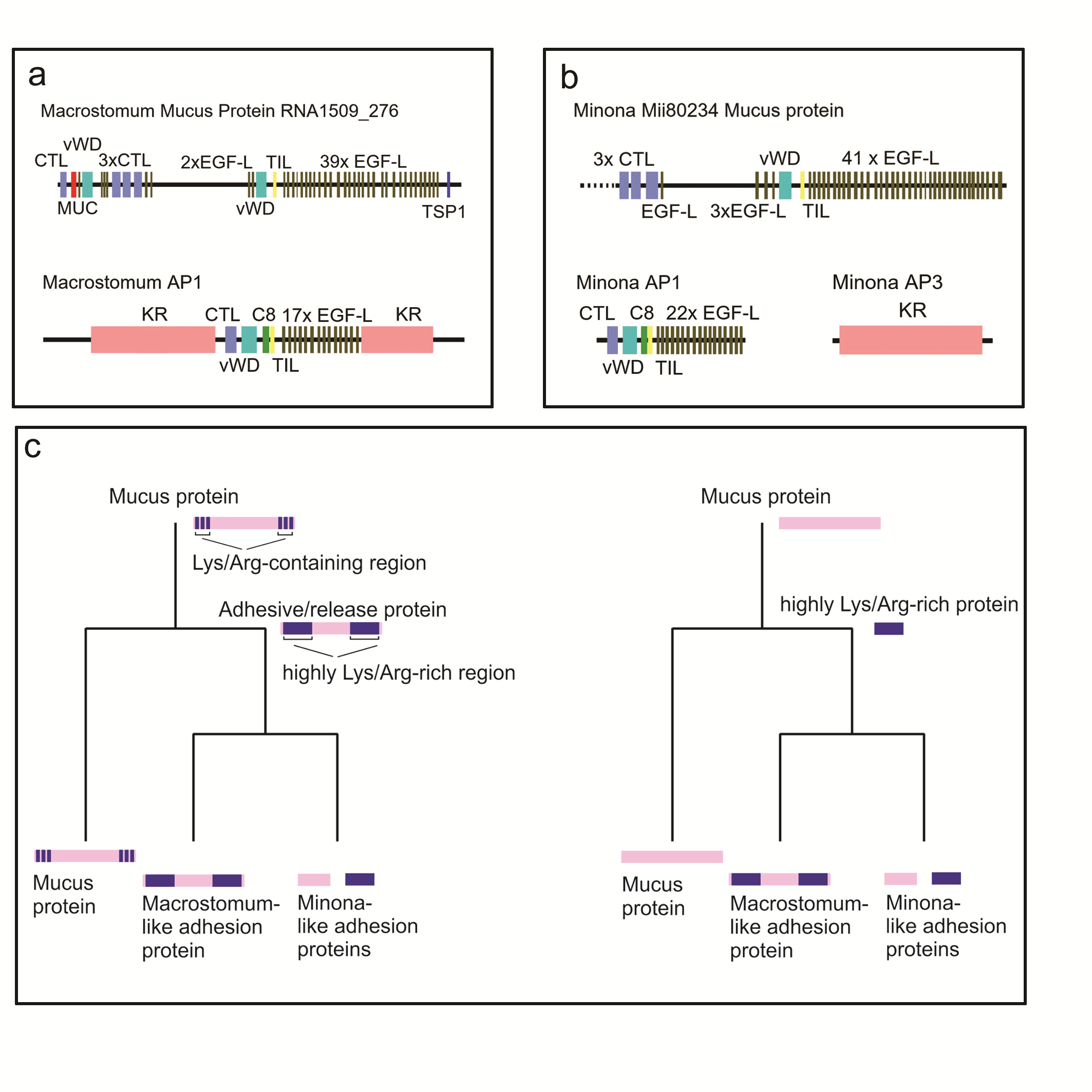


Supplementary figure S13. (*a*) Protein domain organisation of the Macrostomum Mucus protein RNA 1509_276 [1] and the Macrostomum adhesion protein Mlig-ap1. (*b*) Protein domain organisation of the Minona Mii80234 Mucus protein (for sequence see supplementary electronic material, dataset S1) and Minona adhesion protein Mile-ap1. Protein domain abbreviations: C8 domain of 8 conserved cysteines, CTL C-type Lectin domain, EGF-L EGF-like calcium-binding domain, KR Lys/Arg-rich region, MUC Mucin domain, TIL trypsin inhibitor like domain, TSP1 thrombospondin 1 like domain, vWD von-Willebrand-factor type D like domain. (*c*) Evolution of adhesive/release proteins from a mucus ancestor (left) or by acquisition of a KR-rich protein (right). Mucus/adhesion protein (pink), KR-rich regions (blue).

**Supplementary Materials: Tables S1 and S2**

**Supplementary Table S1.**

**Summary of the *M. ileanae* transcriptome assembly.**

| Total number of transcripts | 264 995 |
| --- | --- |
| Number of transcripts after  CD-HIT 98% clustering | 231 117 |
| Total length (bp) | 187 589 403 |
| Longest transcript (bp) | 18 447 |
| Shortest transcript (bp) | 224 |
| Average transcript length (bp) | 707.90 |
| N50 length (bp) | 1 132 |
| Percent GC | 36.40 |

**Supplementary Table S2. Lectins tested in *M. ileanae*.**

| **Lectin** |  | **Specificity** | **Staining** |
| --- | --- | --- | --- |
| UEA I | *Ulex europaeus* Agglutinin I | Fucose, Arabinose | Footprints;Mucus weak |
| PNA | Peanut Agglutinin | Galactose | Footprints; Mucus weak |
| Pha-E | *Phaseolus vulgaris* Erythroagglutinin | Galactose, Complex Structures | Footprints |
| Pha-L | *Phaseolus vulgaris* Leucoagglutinin | Galactose, Complex Structures | Footprints; Mucus |
| GSL I | *Griffonia simplicifolia* Agglutinin I | Galactose, N-Acetylgalactosamine | Footprints weak; Mucus |
| ECL | *Erythrina cristagalli* lectin | Galactose, N-Acetylgalactosamine, Lactose | Footprints; Mucus |
| PSA | *Pisum sativum* agglutinin | Mannose, Glucose | Footprints weak; Mucus weak |
| VVA | *Vicia villosa* agglutinin | N-Acetylgalactosamine | Footprints; Mucus strong |
| GSL II | *Griffonia simplicifolia* Agglutinin II | N-Acetylglucosamine | Footprints; Mucus very weak |
| SNA | *Sambucus nigra* lectin | Sialic Acid | Footprints weak; Mucus weak |
| DBA | *Dolichos biflorus* agglutinin | α-linked N-acetylgalactosamine | Footprints weak; Mucus |
| LEL | *Lycopersicon esculentum* lectin | [GlcNAc]1-3, N-Acetylglucosamine | Bacteria; Mucus very weak |
| STL | *Solanum tuberosum* lectin | N-Acetylglucosamine | Bacteria; Mucus fibres |
| ConA | Concanavalin A | Mannose, Glucose | Mucus |
| MAL II | *Maackia amurensis* lectin | Sialic acid | Mucus |
| SBA | *Glycine max* agglutinin | Galactose, N-Acetylgalactosamine | Mucus |
| JAC | Jacalin | Galactose | Mucus strong |
| RCA I | *Ricinus communis* agglutinin | Galactose, Lactose | Mucus traces |
| WGAs | Wheat germ agglutinin succinylated | N-Acetylglucosamine | Mucus weak |
| DSL | *Datura stramonium* lectin | [GlcNAc]1-3, N-Acetylglucosamine | Mucus weak |
| LCA | *Lens culinaris* agglutinin | Mannose, Glucose | none |
| SJA | *Sophora japonica* lectin | Galactose, N-Acetylgalactosamine | none |
| WGA | Wheat germ agglutinin | N-Acetylglucosamine | none |

**Supplementary Materials and Methods**

**Mass Spectrometry sample preparation and data analysis**

In order to collect footprint proteins, 15 intact *M. ileanae* worms were kept for 30 min in a 1.5 ml protein low-bind tube (Eppendorf) in 50 µl artificial sea water. Three replicate samples were prepared (electronic supplementary material, dataset S2 "footprints intact Animals 1-3"). Likewise, four replicate samples of animals with amputated tails were set up (electronic supplementary material, dataset S2 "footprints of tail amp animals1-4"). *M. ileanae* secreted ample amounts of mucus, which could interfere with the Mass Spectrometry analyses. Therefore another set of five samples consisting of amputated tails only (electronic supplementary material, dataset S2 "footprints of amp tails 1-5") was prepared. Visual examinations confirmed that the tails exhibited attachment- and release behavior. In addition, Lectin staining of footprints of amputated tails confirmed that they secreted adhesive material.

Proteins from flatworm footprints experiments were reduced with 50 µL 10 mM dithiothreitol at 56°C for 30 min and alkylated with 50 µL 55 mM iodoacetamide at room temperature for 20 min. Samples were digested with trypsin at 37°C overnight. The tryptic peptides were purified by ZipTip C18 pipette tips (Millipore) according to the manufacturer’s instructions prior to nanoLC-ESI-MS analysis. Samples were analysed using an UltiMate 3000 nano-HPLC system coupled to a Q Exactive mass spectrometer (both Thermo Scientific). Peptides were separated on a homemade fritless fused-silica microcapillary column (75 μm i.d. × 280 μm o.d. × 10 cm length) packed with 3 μm reversed-phase C18 material (Reprosil). Solvent for HPLC were 0.1% formic acid (solvent A) and 0.1% formic acid in 85% acetonitrile (solvent B).

Data analysis was performed using Proteome Discoverer software (Thermo Scientific) with the search engine Squest against the translated *M. ileanae* transcriptome. Precursor and fragment mass tolerance was set to 10 ppm and 0.02 Da, respectively, and up to two missed cleavages were allowed. Carbamido methylation of cysteine and oxidation of methionine were set as variable modifications. Peptide identifications were filtered at 1% or 5% false discovery rate.

**Transcriptome assembly and differential gene expression analyses**

Illumina paired-end 100 bp sequencing was performed on three libraries generated from three independent RNA isolations from a mixed population of hatchlings, juveniles, and adult animals. We obtained 41 372 986, 34 547 777, and 35 551 263 reads, respectively. Illumina reads were assembled using Trinity v.2.0.6 [2] with default settings. Transcripts were clustered using cd-hit-EST software v.4.5.4 [3, 4]. BUSCO software v.3 [5, 6] was applied to assess the completeness of the transcriptome. There, 978 metazoan orthologues were compared and were considered to be complete (C) in the *M. ileanae* transcriptome if the length of their aligned sequence was within two standard deviations of the BUSCO gene mean length. If this criterion is not met, transcripts were categorized as fragmented (F). If complete genes were present with more than one copy they were classified as duplicated (D). If an ortholog was not found in the transcriptome, it was classified as missing (M).

For the identification of tail-specific transcripts, differential gene expression analyses was performed. Reads from intact animals (70.786.459 50bp reads passing Illumina filter) and amputated worms (83.900.979 50bp reads passing Illumina filter) were aligned to the transcriptome using bwa [7]. Differentially expressed genes (false-discovery rate ≤ 0.05, with a minimum eight fold change) were identified using DESeq2 [8].

**Transmission Electron Microscopy (TEM) and Element Analysis**

Laboratory cultures of *Minona ileanae* were chemically fixed with 2.5% glutaraldehyde in 0.1 M cacodylate buffer containing 10% sucrose, pH 7.3 overnight or longer at 4°C. After rinsing with 0.1 M cacodylate buffer, specimens were post fixed with 1% osmium tetroxide in 0.05 M cacodylate buffer for 1 hour at 4°C, rinsed with buffer, dehydrated in an increasing acetone series and embedded in EMBed812 resin. Additional samples were fixed according to Eisenman and Alfert 1982 [9].

In brief, specimens were prefixed in a cocktail of aldehyde main fixative and 0.05% osmium tetroxide for 10 minutes on ice, fixed for 1 hour in the main fixative at 4°C, washed with buffer and post fixed in the osmium fixative for 1 hour. After rinsing with buffer, specimens were dehydrated and embedded into EMBed812. For high contrast electron microscopy specimens were chemically fixed according as previously described [10]. Sections were cut with a diamond knife (Diatome, Switzerland) on an ultracut UCT (Leica, Austria), stained with lead citrate and examined with a Zeiss Libra 120 energy filter transmission electron microscope (Zeiss, Germany) using zero-loss electrons. Images were made with a 2x2k high speed camera (Tröndle, Germany) and the iTEM software (Olympus, Japan) or the ImageSP software (Tröndle, Germany). RNAi treated animals were fixed with 2.5% glutaraldehyde in 0.1 M cacodylate buffer containing 10% sucrose and post fixed with 1% osmium tetroxide and processed as described above. In addition, specimens were also high pressure frozen with a BALTEC-HPM-010 (Balzers, Lichtenstein), freeze-substituted in acetone supplemented with 1% Osmium tetroxide and 0.2% uranyl acetate by using an AFS2 device (Leica Microsystems, Austria) and embedded into EMBed 812 epoxy resin [11]. Sections were stained with uranyl acetate and/or lead citrate and examined with a CM 120 TEM (Philips/Fisher Thermo Scientific, Netherlands). Images were taken with a MORADA CCD camera (oSIS, Olympus, Japan).

The nitrogen (N) content of the adhesive and releasing granules was measured spectroscopically with electron energy loss spectroscopy (EELS) with a Zeiss Libra 120 Energy Filter Transmission Electron microscope at the maximum element ionization edge at 397 eV. Imaging of the nitrogen distribution was carried out with element spectroscopic imaging (ESI), using a three window method and an inverted high contrast image at 250 eV and mixmapped with false colors. Data analysis and imaging was done with an iTEM software (Olympus, Japan).

**Scanning Electron Microscopy**

Specimens were fixed with 2.5% glutaraldehyde in 0.1 M cacodylate buffer plus sucrose at 4°C for 1 to 2 hours, rinsed with buffer and post fixed in 1% osmium tetroxide in 0.05 M cacodylate for 1 hour at 4°C, dehydrated with a methanol series, critical point dried with a Pelco CPD2 machine (Pelco, USA) and sputtered with gold. Samples were examined with a Zeiss DSM950 SEM (Zeiss, Germany) and images were taken with a Pentax digital camera and a PK Tether 0.7.0 free software.

**Whole mount in situ hybridisation (WISH) and RNA interference**

Whole mount in situ hybridisation was performed in 24 well plates using the *Macrostomum lignano* standard WISH protocol described in previous publications [12, 13] with the following modifications: (1) Animal relaxation using 7.14% MgCl_2_ in H_2_O was extended to 25-30 min. (2) Protease treatment was prolonged to 25 min at 37°C. (3) After rehydration, a bleaching step, routinely done for Planarians [14] with 5% Formamide, 0.5 x SSC, 1.2% H_2_O_2_ under bright light for 2 to 3 hours was introduced. Prior to bleaching two washes with 0,5x SSC buffer and after bleaching three washes with PBS-Tween (0.1%) were performed. Samples were mounted in Mowiol and images were taken using a Leica 5000B Microscope.

RNAi was performed by soaking the animals in ds RNA solution. In brief, animals were cut at the level of the male copulatory bulb (15 per experiment) in order to remove adhesive-organ-related mRNAs and proteins. The anterior parts were transferred to embryo dishes with dsRNA solution () diluted in artificial seawater (final volume 400µl, final dsRNA concentration 1.5 – 2.5 ng/µl). Animals were let to regenerate the tails for six to nine days. dsRNA solution was changed every day. Animals were fed once a day with crushed *Artemia salina* or *Gammarus fossarum*. After regeneration, the animals were checked for their ability to adhere. Additionally, the efficacy of knock-down was evaluated by whole mount *in situ* hybridisation.

**Oxford Nanopore sequencing**

High molecular weight (HMW) DNA was quantified using the Qubit fluorometer with Qubit HS reagents (Thermo Fisher, Waltham, MA, USA) and 1 ng was sized using the Agilent (Santa Clara, CA, USA) Fragment Analyzer System with HS Large Fragment 50 kb Kit (DNF-464) chemistry. A fragment size peak at about 50 kb was observed with a smear ranging from ≈11 to ≈140 kb. This average fragment size was used for subsequent molarity calculations for library preparation. The HS Large Fragment Kit had been previously qualified using conventional pulsed-field electrophoresis on a BioRad (USA) Chef Mapper System.

To maximize the read lengths, no DNA fragmentation was performed prior to library preparation. Based on previous experiences with HMW DNA, the two magnetic beads elution steps in the protocol were prolonged to 10 and 30 min, respectively, to enhance the elution efficiency of very high molecular DNA molecules. The LSK108 protocol requires loading 5 to 50 fmol of library molecules on the flow cell for optimal sequencing. However, a loss of 60-80% of HMW input material during library preparation steps is commonly seen. Therefore, we started the library preparation with a total amount of 3.2 µg DNA, following the rationale that this shall result in 0.64 µg to 1.28 µg of library after the expected losses. This amount corresponds to 19 to 39 fmol at 50 kb size, which is well within the optimal range. Finally, 489.6 ng of sequencing library (15 fmol) were loaded on the flow cell and a 48 hour sequencing run on a R9.4 (FLO-MIN106) flow cell was performed.

Base calling was performed online using the ONT MinKNOW Software (v1.14.1), resulting in 6.25 GB (776,590 reads) of data passing the basecaller quality filter. Output and quality statistics were generated using the *NanoPack* package [15] v1.18.2 (https://github.com/wdecoster/nanopack). Since the *NanoQC* tool (a part of *NanoPack*) indicated deteriorating read quality and unexpected fluctuations in base distribution at the beginning and end of the reads, the first 40 and last 12 bases of each read were cropped using the *Trimmomatic* tool v0.38 [16] to generate the final analysis dataset.

**Flow cytometric measurement of genome size**

We used a detergent-trypsin method with propidium iodide (PI) staining for flow cytometric analysis of genome size [17]. Starved worms were washed in few millilitres of stock solution (3.4 mM Trisodium citrate dihydrate, Nonidet P40 at 0.1% v/v, 1.5 mM Sperminetetrahydrochloride, 0.5 mM Trishydroxymethylaminomethane, pH 7.6), and were then transferred to 750 μl stock solution in a 1 ml-dounce tissue homogenizer. For each replicate, we used 3-6 worms, which were homogenized on ice using the “tight” pestle of the homogenizer, together with an internal standard of known genome size. These internal standards were: (i) the fruit fly, *Drosophila melanogaster* (strain ISO-1, 1C-value: 0.18 pg, Gregory 2019), a clone of the rotifer *B. asplanchnoidis* (clone ohj72, 1C-value: 0.402 pg, [18]), and chicken erythrocyte nuclei (CEN, 1C-value: 1.25 pg [19]). Homogenization was performed with 35 strokes for worms and rotifers, while two female *Drosophila* heads were added to the homogenizer just before the last 10 strokes. For CEN, we used a nuclei suspension provided in a commercial kit (DNA QC Particles, BD biosciences). Large debris was removed by filtration through a 40 μm mesh nylon sieve. After addition of 100 μl of 0.021% Trypsin (dissolved in stock solution) the sample was incubated for exactly 10 min at 37°C. To prevent further degradation, 75µl of 0.25% trypsin inhibitor was added (this solution also included 0.05% RNAse A) and the samples were incubated for another 10 min at 37°C. Finally, samples were stained with propidium iodide at a concentration of 50 μg/ml. Stained samples were kept overnight on ice in the dark. Flow cytometric analysis was performed on the next day on an Attune NxT^®^ acoustic focusing cytometer (Thermo Fisher) with an excitation wavelength of 561 nm (yellow) and a custom-made 590–650 nm bandpass filter for detection of PI fluorescence. Flow cytometric data were analysed using FlowJo software version 10.0.7r2 (FlowJo LLC). Coefficients of variance (CVs) of individual peaks were <4% for all samples, in most cases around 2%. Conversion from picograms DNA to base pairs were made with the factor: 1 pg =978 Mbp [19].

References

[1] Grudniewska, M., Mouton, S., Simanov, D., Beltman, F., Grelling, M., de Mulder, K., Arindrarto, W., Weissert, P. M., van der Elst, S. & Berezikov, E. 2016 Transcriptional signatures of somatic neoblasts and germline cells in Macrostomum lignano. *Elife* **5**. (DOI:10.7554/eLife.20607).

[2] Haas, B. J., Papanicolaou, A., Yassour, M., Grabherr, M., Blood, P. D., Bowden, J., Couger, M. B., Eccles, D., Li, B., Lieber, M., et al. 2013 De novo transcript sequence reconstruction from RNA-seq using the Trinity platform for reference generation and analysis. *Nat Protoc* **8**, 1494-1512. (DOI:10.1038/nprot.2013.084).

[3] Li, W. & Godzik, A. 2006 Cd-hit: a fast program for clustering and comparing large sets of protein or nucleotide sequences. *Bioinformatics* **22**, 1658-1659. (DOI:10.1093/bioinformatics/btl158).

[4] Fu, L., Niu, B., Zhu, Z., Wu, S. & Li, W. 2012 CD-HIT: accelerated for clustering the next-generation sequencing data. *Bioinformatics* **28**, 3150-3152. (DOI:10.1093/bioinformatics/bts565).

[5] Simao, F. A., Waterhouse, R. M., Ioannidis, P., Kriventseva, E. V. & Zdobnov, E. M. 2015 BUSCO: assessing genome assembly and annotation completeness with single-copy orthologs. *Bioinformatics* **31**, 3210-3212. (DOI:10.1093/bioinformatics/btv351).

[6] Waterhouse, R. M., Seppey, M., Simao, F. A., Manni, M., Ioannidis, P., Klioutchnikov, G., Kriventseva, E. V. & Zdobnov, E. M. 2017 BUSCO applications from quality assessments to gene prediction and phylogenomics. *Mol Biol Evol*. (DOI:10.1093/molbev/msx319).

[7] Li, H. & Durbin, R. 2009 Fast and accurate short read alignment with Burrows-Wheeler transform. *Bioinformatics* **25**, 1754-1760. (DOI:10.1093/bioinformatics/btp324).

[8] Love, M. I., Huber, W. & Anders, S. 2014 Moderated estimation of fold change and dispersion for RNA-seq data with DESeq2. *Genome Biol* **15**, 550. (DOI:10.1186/s13059-014-0550-8).

[9] Eisenman, E. A. & Alfert, M. 1982 A New Fixation Procedure for Preserving the Ultrastructure of Marine Invertebrate Tissues. *J Microsc-Oxford* **125**, 117-120. (DOI:DOI 10.1111/j.1365-2818.1982.tb00327.x).

[10] Deerinck, T. J., Bushong, E., Thor, A. & Ellisman, M. 2010 *NCMIR methods for 3D EM: A new protocol for preparation of biological specimens for serial block face scanning electron microscopy*6-8 p.

[11] Salvenmoser, W., Egger, B., Achatz, J. G., Ladurner, P. & Hess, M. W. 2010 Electron microscopy of flatworms standard and cryo-preparation methods. *Methods in cell biology* **96**, 307-330. (DOI:10.1016/s0091-679x(10)96014-7).

[12] Lengerer, B., Pjeta, R., Wunderer, J., Rodrigues, M., Arbore, R., Scharer, L., Berezikov, E., Hess, M. W., Pfaller, K., Egger, B., et al. 2014 Biological adhesion of the flatworm *Macrostomum lignano* relies on a duo-gland system and is mediated by a cell type-specific intermediate filament protein. *Front Zool* **11**, 12. (DOI:10.1186/1742-9994-11-12).

[13] Pfister, D., De Mulder, K., Hartenstein, V., Kuales, G., Borgonie, G., Marx, F., Morris, J. & Ladurner, P. 2008 Flatworm stem cells and the germ line: developmental and evolutionary implications of macvasa expression in Macrostomum lignano. *Dev Biol* **319**, 146-159. (DOI:10.1016/j.ydbio.2008.02.045).

[14] King, R. S. & Newmark, P. A. 2013 In situ hybridization protocol for enhanced detection of gene expression in the planarian Schmidtea mediterranea. *BMC Dev Biol* **13**, 8. (DOI:10.1186/1471-213X-13-8).

[15] De Coster, W., D'Hert, S., Schultz, D. T., Cruts, M. & Van Broeckhoven, C. 2018 NanoPack: visualizing and processing long-read sequencing data. *Bioinformatics* **34**, 2666-2669. (DOI:10.1093/bioinformatics/bty149).

[16] Bolger, A. M., Lohse, M. & Usadel, B. 2014 Trimmomatic: a flexible trimmer for Illumina sequence data. *Bioinformatics* **30**, 2114-2120. (DOI:10.1093/bioinformatics/btu170).

[17] Stelzer, C. P., Riss, S. & Stadler, P. 2011 Genome size evolution at the speciation level: The cryptic species complex Brachionus plicatilis (Rotifera). *Bmc Evolutionary Biology* **11**.

[18] Michaloudi, E., Mills, S., Papakostas, S., Stelzer, C. P., Triantafyllidis, A., Kappas, I., Vasileiadou, K., Proios, K. & Abatzopoulos, T. J. 2017 Morphological and taxonomic demarcation of Brachionus asplanchnoidis Charin within the Brachionus plicatilis cryptic species complex (Rotifera, Monogononta). *Hydrobiologia* **796**, 19-37.

[19] Gregory, T. R. 2109 Animal Genome Size Database. <http://www.genomesize.com>.
